# Harnessing 3D collagen hydrogel-directed conversion of human GMSCs into SCP-like cells to generate functionalized nerve conduits

**DOI:** 10.1038/s41536-021-00170-y

**Published:** 2021-09-30

**Authors:** Qunzhou Zhang, Phuong Nguyen, Justin C. Burrell, Jincheng Zeng, Shihong Shi, Rabie M. Shanti, Grace Kulischak, D. Kacy Cullen, Anh D. Le

**Affiliations:** 1 Department of Oral and Maxillofacial Surgery and Pharmacology, University of Pennsylvania School of Dental Medicine, 240 South 40th Street, Philadelphia, PA USA; 2 Division of Pediatric Plastic Surgery, the University of Texas Health Science Center at Houston, 6431 Fannin Street, Houston, TX USA; 3 Department of Neurosurgery, University of Pennsylvania Perelman School of Medicine, 3320 Smith Walk, Philadelphia, PA USA; 4 Center for Neurotrauma, Neurodegeneration, and Restoration, Corporal Michael J. Crescenz VA Medical Center, 3900 Woodland Ave, Philadelphia, PA USA; 5 Guangdong Provincial Key Laboratory of Medical Molecular Diagnostics, Guangdong Key Laboratory of Medical Bioactive Molecular Developmental and Translational Research, Guangdong Medical University, Dongguan, China; 6 Department of Oral & Maxillofacial Surgery, Penn Medicine Hospital of the University of Pennsylvania, Perelman Center for Advanced Medicine, 3400 Civic Center Blvd, Philadelphia, PA USA

**Keywords:** Stem-cell research, Regeneration

## Abstract

Achieving a satisfactory functional recovery after severe peripheral nerve injuries (PNI) remains one of the major clinical challenges despite advances in microsurgical techniques. Nerve autografting is currently the gold standard for the treatment of PNI, but there exist several major limitations. Accumulating evidence has shown that various types of nerve guidance conduits (NGCs) combined with post-natal stem cells as the supportive cells may represent a promising alternative to nerve autografts. In this study, gingiva-derived mesenchymal stem cells (GMSCs) under 3D-culture in soft collagen hydrogel showed significantly increased expression of a panel of genes related to development/differentiation of neural crest stem-like cells (NCSC) and/or Schwann cell precursor-like (SCP) cells and associated with NOTCH3 signaling pathway activation as compared to their 2D-cultured counterparts. The upregulation of NCSC-related genes induced by 3D-collagen hydrogel was abrogated by the presence of a specific NOTCH inhibitor. Further study showed that GMSCs encapsulated in 3D-collagen hydrogel were capable of transmigrating into multilayered extracellular matrix (ECM) wall of natural NGCs and integrating well with the aligned matrix structure, thus leading to biofabrication of functionalized NGCs. In vivo, implantation of functionalized NGCs laden with GMSC-derived NCSC/SCP-like cells (designated as GiSCs), significantly improved the functional recovery and axonal regeneration in the segmental facial nerve defect model in rats. Together, our study has identified an approach for rapid biofabrication of functionalized NGCs through harnessing 3D collagen hydrogel-directed conversion of GMSCs into GiSCs.

## Introduction

Mesenchymal stromal/stem cells (MSCs), a subpopulation of post-natal stem cells existing in almost all mesodermal connective tissues^1^, possess multipotent differentiation capabilities, potent immunomodulatory/anti-inflammatory functions, and pleiotropic effects through the secretion of a large panel of growth factors, making them a good candidate for cell-based therapies to treat a large spectrum of diseases or pathological conditions^2,3^, including peripheral nerve regeneration^4,5^. In recent years, a similar subpopulation of MSCs endowed with neural crest stem-like cell (NCSC) properties have been isolated and identified from various neural crest (NC)-derived adult tissues, such as dental pulp^6^, oral mucosa/gingiva^7–9^, bone marrow^10,11^, and skin epidermis^12^. Following in vitro culture and expansion, the NCSC properties are lost resulting in differentiation into heterogeneous MSCs. Therefore, special culture conditions (e.g., crestosphere), are required to maintain their NCSC properties^6–8,13^. Due to their wide existence and multipotency, adult NCSCs, particularly those from the easily accessible orofacial tissues^14^, represent an attractive source of stem cells for cell-based regenerative therapy of various diseases, particularly, for nerve regeneration because of their intrinsic propensity to differentiate into glial and Schwann-like cells^15–17^.

In recent years, the advancement in tissue engineering (TE) technology, stem cell biology, and biomaterial science have directed increasing efforts to fabricate the next generation of nerve guidance conduits (NGC) or tissue-engineered medical products by combining the use of different types of scaffolds, growth factors and/or different types of supportive cells^18^, among which primary Schwann cells or Schwann-like cells induced from pluripotent or MSCs could be the optimal type of supportive cells due to their critical role in orchestrating nerve repair/regeneration process^15^. However, it is challenging to obtain a sufficient number of primary Schwann cells due to the limited availability and difficulty in culturing and expansion^19^. Meanwhile, the efficiency of transdifferentiating mesodermal MSCs into Schwann-like cells varies greatly due to the heterogeneity caused by various intrinsic and extrinsic factors^5,19^. Recent studies have shown that somatic cells, such as fibroblasts^20–23^ and epidermal keratinocytes^24^, can be directly converted into NCSC- or Schwann cell precursor (SCP)-like cells^25,26^ via introduction of a single/multiple transcriptional factors or non-genetic approaches, such as defined culture conditions, the use of a cocktail of small molecules, and tuning the mechanical properties (pore size, stiffness, etc.) of natural or synthetic biomaterials^20,24,26–28^.

In traditional scaffold-based TE, supportive cells can be delivered by incorporation/encapsulation into the scaffolds or seeding on the surface of engineered scaffolds post-fabrication^29–32^. Cell incorporation/encapsulation before fabrication allows the formation of a high cell density within the scaffolds, but it is difficult to achieve homogeneous cell distribution, good patterning or layering of cells within the scaffolds; cell seeding methods post-fabrication can minimize cell damage caused by the harsh fabrication conditions, but the efficiency of cell seeding and penetration is also a significant issue even with the use of a bioreactor^29,30^. In terms of tissue engineering nerve grafts or NGCs, either through conventional scaffold-based TE or 3D bioprinting, supportive cells combined with a certain type of scaffold can be fabricated either as the filler of a hollow NGC or loaded into the wall of a NGC^29,33^. From the standpoint of clinical translation, techniques utilized to fabricate cell-based TE products should be efficient, rapid, reproducible, and easy to use in order to minimize cellular damage and maximize a homogeneous distribution within the scaffolds^34^. However, even the novel 3D bioprinting technology has several challenges to achieve such translational goals^30^.

In the present study, we harnessed the property of methacrylated 3D-collagen hydrogel to convert NC-derived human gingiva-derived mesenchymal stem cells (GMSCs) into NCSC/SCP-like cells (designated as GiSCs), which were capable of migrating into the wall matrix of natural nerve conduits, thus leading to generation of functionalized NGCs for treatment of segmental facial nerve injury in rats.

## Results

### 3D-collagen hydrogel drives the conversion of GMSCs into NCSC/SCP-like cells

According to our previous studies^35^, human gingiva-derived mesenchymal stem cells (GMSCs) were routinely isolated and characterized by the expression of several MSC-associated cell surface markers, e.g., CD44, CD73, and CD90, but negative for hematopoietic cell markers, e.g., CD45 (Supplementary Fig. 1a, b) as well as their multipotent differentiation capacities into adipocytes (Supplementary Fig. 1c, d) and osteocytes (Supplementary Fig. 1e, f).

**Fig. 1:**
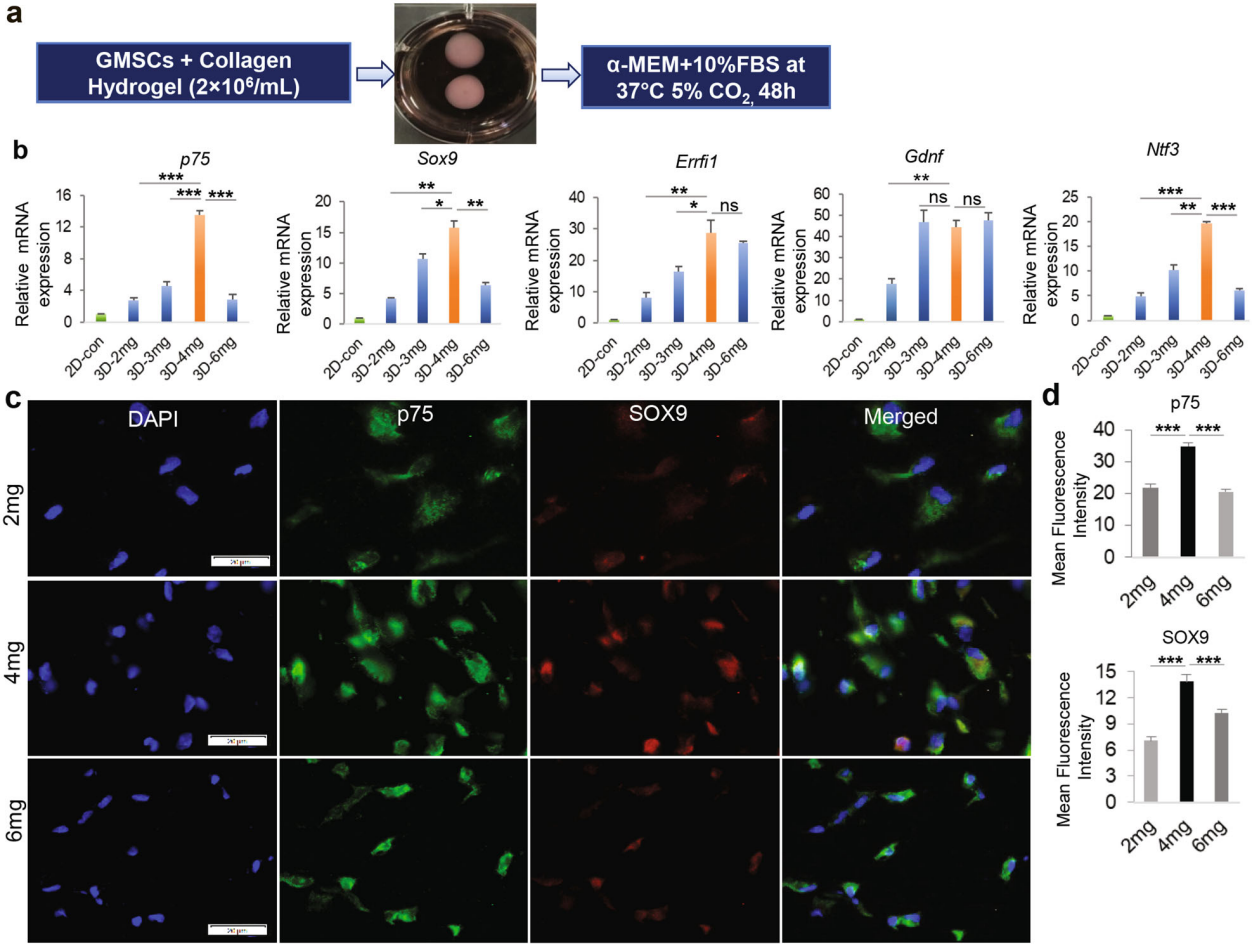
Upregulation of NCSC/SCP-related genes in GMSCs cultured in methacrylated 3D-collagen hydrogel. **a** GMSCs (2 × 10^6^/mL) were encapsulated in 3D-collagen hydrogel at different concentrations (2, 3, 4, 6 mg/mL) and cultured in complete α-MEM for 48 h. **b** The expression levels of *Ngfr* (p75), *Sox9*, *Errfi1*, *Gdnf*, and *Ntf3* in GMSCs cultured in 3D collagen hydrogel were determined by qRT-PCR as compared to those in 2D-cultured GMSCs (2D-con). **c** Immunofluorescence (IF) staining against p75 (green color) and SOX9 (red color) in cryosections of 3D-collagen hydrogel encapsulated with GMSCs. Nuclei were counterstained with 4′, 6-diamidino-2-phenylindole (DAPI; white or blue). Scale bar = 20 µm (**c**). **d** Quantification of IF intensity of p75 and SOX9. Data represented the mean ± SD, *n* = 3 biological replicates. *p < 0.05; **p < 0.01; ***p < 0.001; ns, no significant; one-way ANOVA with the Tukey’s post test (**b**, **d**). 2D-con, GMSCs cultured in 2D-conditions; 3D, GMSCs cultured in 3D-collagen hydrogel with different concentrations.

**Fig. 2:**
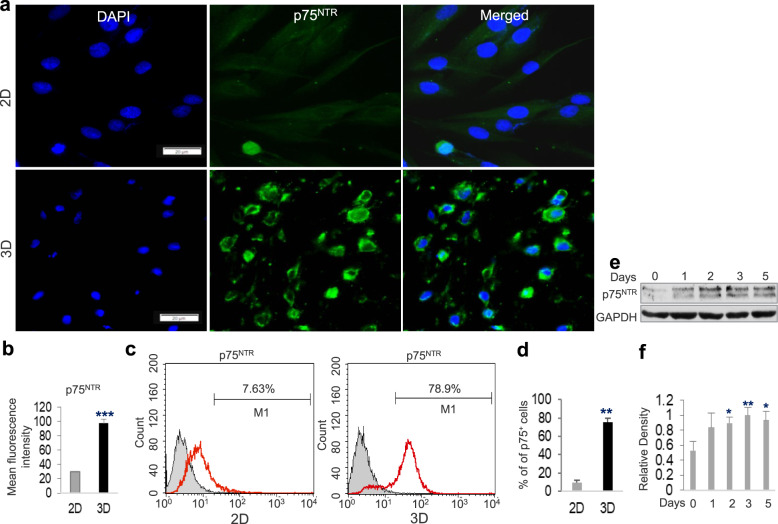
Increased expression of p75^NTR^ protein in GMSCs cultured in methacrylated 3D-collagen hydrogel. GMSCs were cultured under 2D cultures or in 3D-collagen hydrogel (at 4 mg/ml) in complete α-MEM for 48 h. **a** Immunofluorescence staining against p75^NTR^ (green color), while nuclei were counterstained with 4′, 6-diamidino-2-phenylindole (DAPI; blue). Scale bar = 20 µm. **b** Quantification of IF intensity of p75. **c** GMSCs were harvested from 2D-culture or recovered from 3D collagen hydrogel via digestion with collagenase I and then immunofluorescence staining for p75^NTR^, followed by incubation with Alexa Fluor 488-conjugated secondary antibodies. The cell samples were analyzed by a flow cytometer (FCM). **d** The average of p75^+^ cells from FCM analysis. **e** GMSCs encapsulated 3D collagen hydrogel were cultured for different time periods and the expression of p75^NTR^ protein was determined by Western blot (WB). **f** Quantification of the relative WB density of p75^NTR^ with GAPDH as the internal control. Data represent the mean ± SD, *n* = 3 biological replicates. *p < 0.05; **p < 0.01; ***p < 0.001; ns no significant; Student’s two-tailed unpaired *t*-test (**b**, **d, f**). 2D, GMSCs cultured in 2D-conditions; 3D, GMSC cultured in 3D-collagen hydrogel.

Several lines of evidence have shown that the physical properties of the 3D-scaffolds or substrate, e.g., its porosity and stiffness (a combined single parameter known as matrix density) can mechanically influence phenotypic conversion or differentiation of stem cells toward a special cell lineage^36,37^. Herein, we initially cultured GMSCs for 48 h in methacrylated 3D-collagen hydrogel with different matrix densities or stiffness achieved by varying collagen concentrations (2, 3, 4, 6 mg/mL) with regular MSC culture medium (α-MEM + 10% FBS) (Fig. 1a), and then mRNA expression of NCSC/SCP-related genes was determined by qRT-PCR. Unexpectedly, our results showed that GMSCs cultured in collagen gel at 4 mg/mL had the largest increase in expression of *p75*^NTR^, *Sox9*, ERBB Receptor Feedback Inhibitor 1 (*Errfi1*), and glial cell-derived neurotrophic factor (*Gdnf*) as compared to those in 2D-cultured GMSCs (Fig. 1b). An optimal increase in the protein expression of p75^NTR^ (NGFR) and SOX9, two common NCSC/SCP-related genes^24,38^, was confirmed in GMSCs cultured in 3D collagen hydrogel at a concentration of 4 mg/mL (Fig. 1c, d). Based on these findings, collagen hydrogel with a concentration of 4 mg/mL was selected as the optimal matrix density for all the subsequent studies. The increased expression of p75^NTR^ protein in GMSCs cultured in 3D-collagen hydrogel at 4 mg/mL was further confirmed by IF staining (Fig. 2a, b). Flow cytometric analysis indicated that about 80% of 3D-cultured GMSCs are positive for p75^NTR^ compared to 7.6% in 2D-cultured GMSCs (Fig. 2c, d). Western blot showed that the expression of p75^NTR^ protein started to increase at day 1 in GMSCs after cultured in 3D-collagen hydrogel, which was maintained up to day 5 (Fig. 2e, f). Of note, when 2D plastic culture dishes were pre-coated with 4 mg/mL methacrylated collagen hydrogel and then GMSCs were seeded onto the surface of the solidified hydrogel (Supplementary Fig. 2a) and cultured for 48 h, cells exhibited a more elongated morphology than those encapsulated in the hydrogel (Supplementary Fig. 2b). Meanwhile, GMSCs seeded on top of the hydrogel did not show an increased expression of p75^NTR^ (Supplementary Fig. 2c). In addition, our results indicated that human bone marrow-derived mesenchymal stem cells (hBMSCs), as characterized by the expression of MSC-associated cell surface markers (Supplementary Fig. 3a) and adipogenic/osteogenic differentiation potentials (Supplementary Fig. 3b), didn’t show an increased expression of p75^NTR^ and SOX9 when encapsulated in the methacrylated 3D-collagen hydrogel and cultured under the same culture conditions as GMSCs (Supplementary Fig. 3c). These results suggest that GMSCs encapsulated in methacrylated 3D-collagen hydrogel with an optimal stiffness could be converted toward a NCSC/SCP-like phenotype.

### Gene expression profiling by the next-generation RNA-seq

To further characterize the phenotypic changes in GMSCs cultured under 3D-collagen hydrogel, the next- generation RNA-sequencing was performed to profile the gene expression patterns in GMSCs cultured under 2D-monolayer and 3D-collagen hydrogel conditions, whereby significant differentially expressed genes (DEGs) were defined as those with a log2 fold change (FC) ≥1 (GMSC-3D v.s.GMSC-2D) and a false discovery rate (FDR) of ≤1%. A total of 5588 DEGs, including 3476 upregulated and 2112 downregulated genes were identified from GMSCs cultured in the 3D collagen hydrogel compared to 2D-cultured GMSCs (Supplementary Fig. 4a). Hierarchical cluster analysis revealed a different hierarchical clustering algorithm in 3D- and 2D-cultured GMSCs as illustrated in the heatmap (Supplementary Fig. 4b). These findings suggest that GMSCs cultured in 3D-collagen hydrogel underwent significant transcriptome changes as compared with those under regular 2D-culture conditions.

### Gene functional annotation/classification of DEGs in GMSCs cultured in 3D-collagen hydrogel

**Fig. 3:**
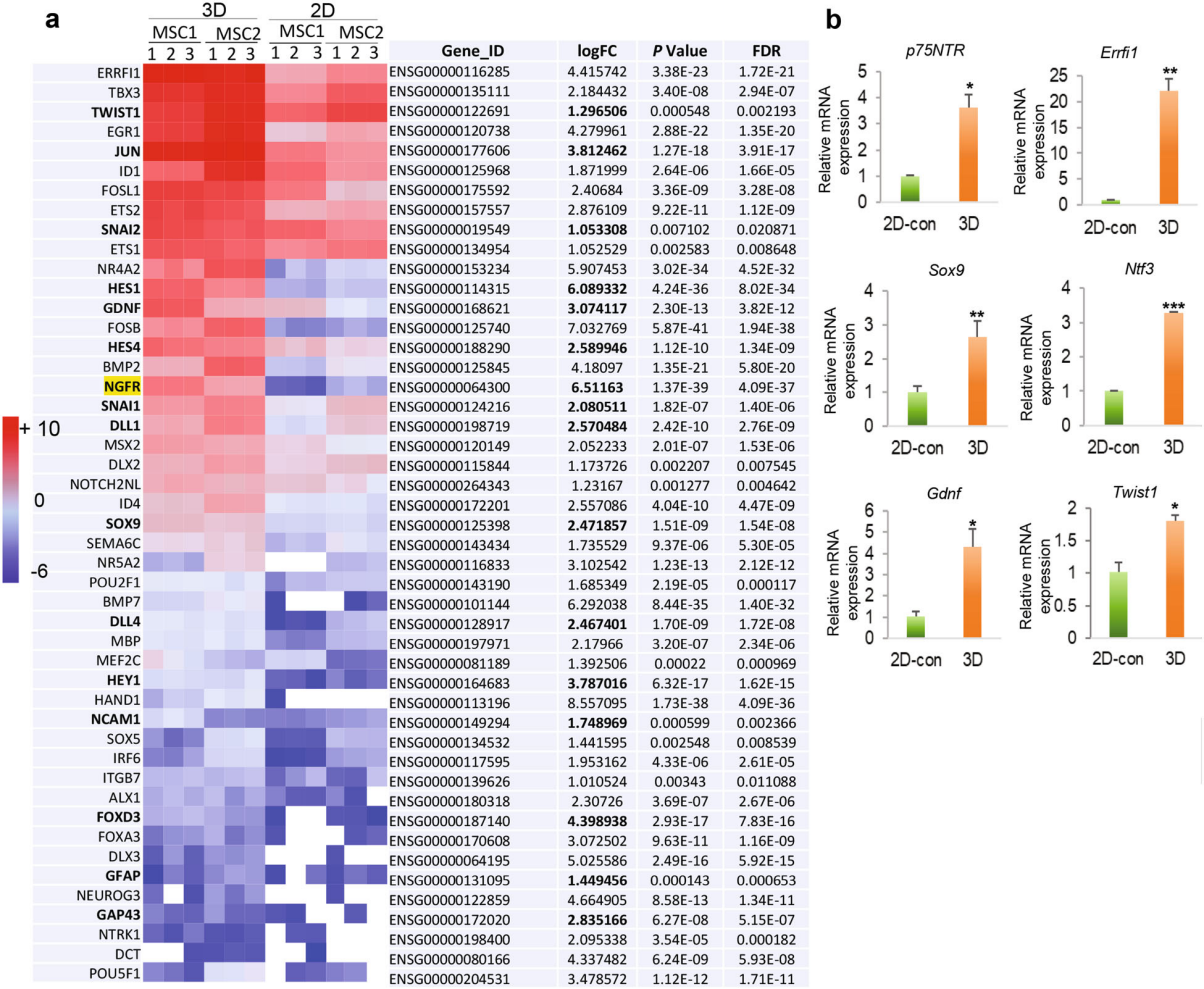
RNA-seq on the expression profile of neural crest and Schwann cell precursor cell-related genes in GMSCs cultured in methacrylated 3D-collagen hydrogel. GMSCs were encapsulated in 3D-collagen hydrogel (4 mg/mL) at a cell density of 2 × 10^6^/mL and cultured in complete α-MEM for 48 h. Total RNA was extracted from 2D- and 3D-cultured GMSCs for next-generation *RNA-seq* or qRT-PCR. **a** Heatmap illustrates NCSC/SCP-like cell-related genes that are significantly upregulated across all samples of GMSCs cultured in 3D collagen hydrogel as compared to the paired 2D-cultured GMSCs (triplicates in two-pairs of GMSCs). The red represents high expression and blue represents low expression. logFC, log2 (fold change; 3D-cultured GMSCs over those 2D-cultured). **b** The upregulation of several NCSC-related genes in GMSCs cultured in 3D-collagen hydrogel was confirmed by quantitative RT-PCR as compared to those in 2D-cultured GMSCs as controls (2D-con). Data represent the mean ± SD, *n* = 3 biological replicates. *p < 0.05; **p < 0.01; ***p < 0.001. Student’s two-tailed unpaired *t*-test (**b**). 2D or 2D-con, GMSCs cultured in 2D-conditions; 3D, GMSC cultured in 3D-collagen hydrogel.

Through DAVID gene functional annotation/classification of those significantly upregulated DEGs in 3D-cultured GMSCs, we identified 47 genes related to specification and function of NCSC and/or SCPs (Fig. 3a)^39^, which include cell surface markers such as NGFR (p75^NTR^), growth factors such as GDNF, transcription factors such as *TBX3*, *TWIST*, *JUN*, *SNAI2* (Slug), *SNAIL1*, *ETS1*, *ETS2*, *ID1*, and *SOX9*, and Notch signaling (Fig. 3a). Among these upregulated genes, the mRNA expression levels of several genes, including *p75*^NTR^, *Sox9*, *Errfi1*, *Gdnf*, *Ntf3*, and *Twist1*, were further confirmed by qRT-PCR (Fig. 3b).

**Fig. 4:**
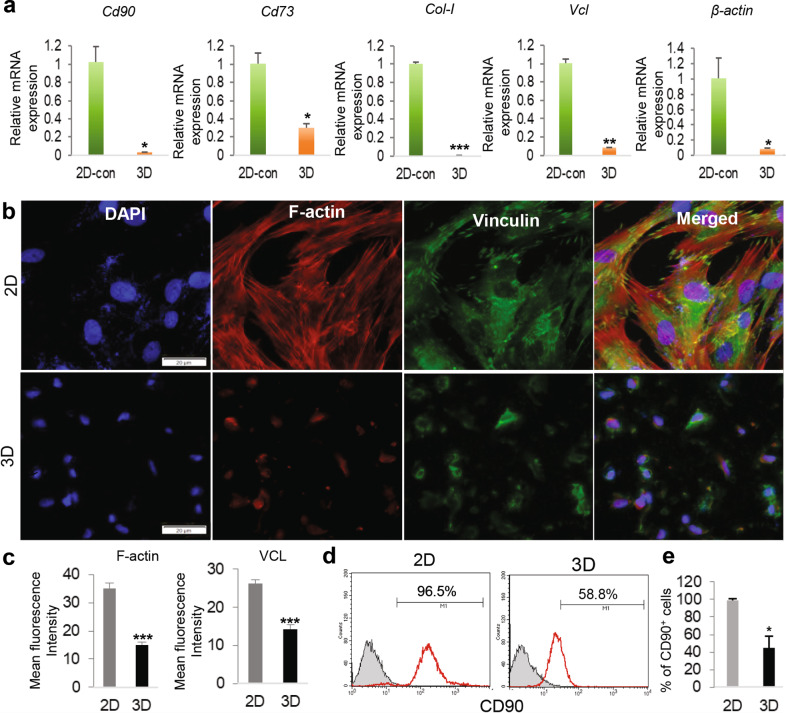
Downregulation of mesenchymal cell-associated marker genes in GMSCs cultured in methacrylated 3D-collagen hydrogel. **a** GMSCs were encapsulated in 3D-collagen hydrogel (4 mg/mL) at a cell density of 2 × 10^6^/mL and cultured in complete α-MEM for 48 h. The mRNA expression of mesenchymal cell-associated genes, *Cd90 (Thy1)*, *Cd73*, *Col-I*, *Vcl*, and *β-actin*, in 3D-cultured GMSCs was determined by quantitative RT-PCR as compared to those in 2D-cultured GMSCs. **b** GMSCs were cultured under 2D culture or in 3D-collagen hydrogel for 48 h. Immunofluorescence (IF) staining for vinculin (green color) while the cytoskeleton (F-actin) was stained with tetramethylrhodamine (TRITC)-conjugated phalloidin, a high-affinity F-actin probe (red). Nuclei were counterstained with 4′, 6-diamidino-2-phenylindole (DAPI; blue). Scale bar = 20 µm. **c** Quantification of IF intensity of vinculin (VCL) and F-actin. **d** GMSCs were harvested from 2D-culture or recovered from 3D collagen hydrogel via digestion with collagenase I and then immunostained with specific antibodies for CD90 (THY1), followed by incubation with Alexa Fluor 488-conjugated secondary antibodies. The cell samples were analyzed by a flow cytometer (FCM). **e** The average of CD90^+^ cells from FCM analysis. Data represent the mean ± SD, *n* = 3 biological replicates. *p < 0.05; **p < 0.01; ***p < 0.001. Student’s two-tailed unpaired *t*-test (**a, c, e**). Col-I, type I collagen; Vcl, vinculin; 2D or 2D-con, GMSCs cultured in 2D-conditions; 3D, GMSC cultured in 3D-collagen hydrogel.

MSCs were characterized by the expression of a panel of mesenchymal markers such as CD73, CD90, and type I collagen^40^. Previous studies indicated that MSCs showed decreased expression of cell adhesion molecules, e.g., vinculin, and cytoskeletal proteins, e.g., F-actin, when cultured in soft substrate^41^. Our recent study showed that GMSCs gradually reduced the expression of MSC-associated cell surface markers during nongenetic induction into NCSC-like cells^42^. Herein, our results indicated that GMSCs cultured in 3D-collagen hydrogel showed a significant decrease in the mRNA expression of mesenchymal genes, such as type I collagen (*ColI*), vinculin (*VCL*), *β-actin*, *Cd90*, and *Cd73*, as determined by qRT-PCR (Fig. 4a). Meanwhile, GMSCs cultured in 3D-collagen hydrogel underwent morphological changes including reduced cell volume, nuclear size, and relaxation of the cytoskeleton (Fig. 4b). The decreased expression of VCL and F-actin at the protein level was further confirmed by immunofluorescence staining (Fig. 4b, c), while the decreased protein expression of CD90 in GMSCs cultured in 3D-collagen hydrogel was confirmed by flow cytometric analysis (Fig. 4d, e). Of note, our results indicated that GMSCs recovered from 3D-collagen hydrogel lost their multipotent differentiation capacities into adipocytes (Supplementary Fig. 1c, d) and osteocytes (Supplementary Fig. 1e, f). Taken together, these findings further support that GMSCs cultured in 3D collagen hydrogel lost their mesenchymal properties.

### Upregulation of NOTCH3 signaling pathway in GMSCs cultured in 3D-collagen hydrogel

**Fig. 5:**
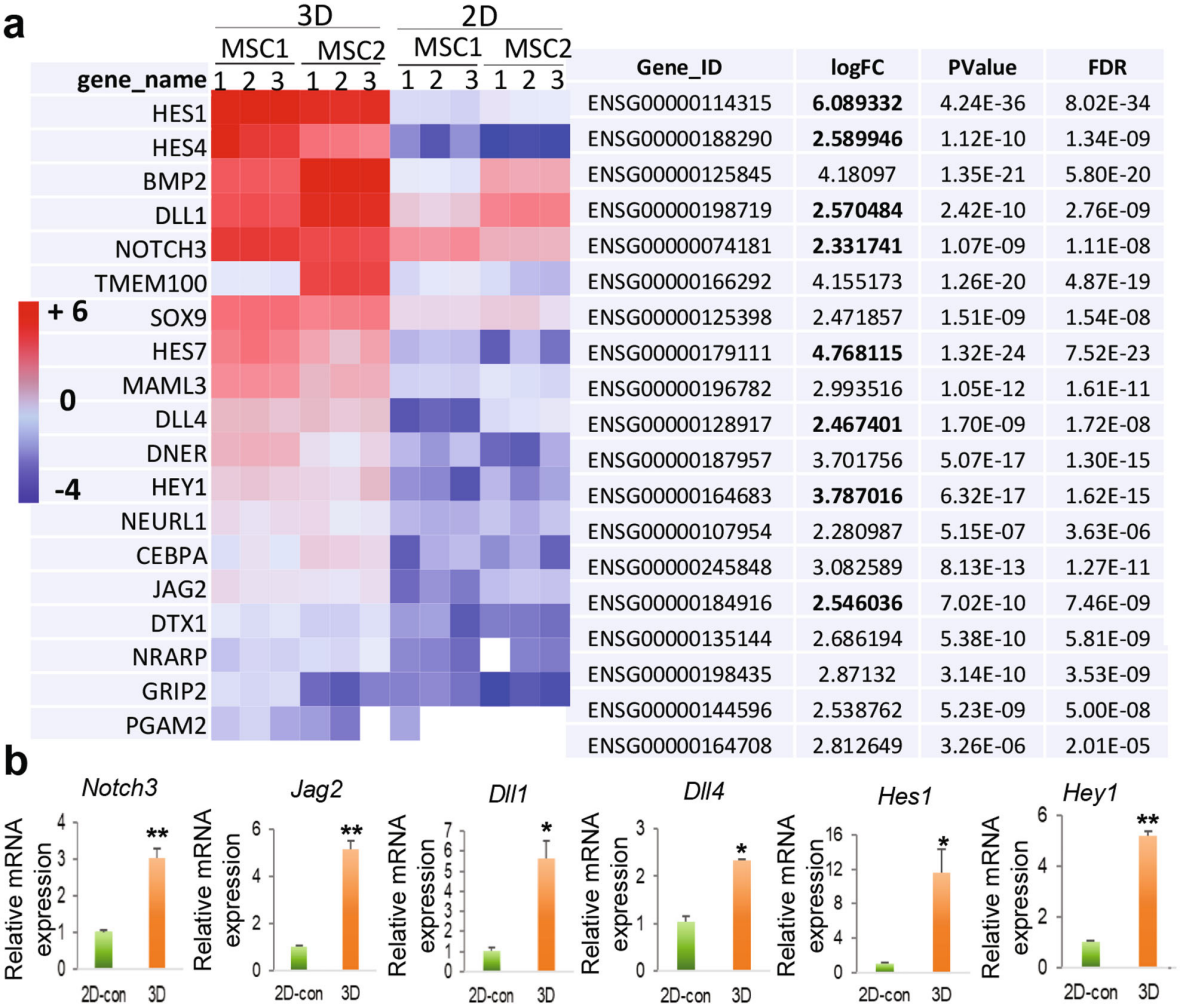
Upregulated mRNA expression of a cluster of Notch signaling-related genes in GMSCs cultured in methacrylated 3D-collagen hydrogel. GMSCs were encapsulated in 3D-collagen hydrogel (4 mg/mL) at a cell density of 2 × 10^6^/mL and cultured in complete α-MEM for 48 h. Total RNA was extracted from 2D- and 3D-cultured GMSCs for RNA-*seq* or qRT-PCR. **a** Heatmap illustrates genes related to Notch signaling pathway that are significantly upregulated across all samples of GMSCs cultured in 3D collagen hydrogel as compared to the paired 2D-cultured GMSCs (triplicates in two-pairs of GMSCs). The red represents high expression and blue represents low expression. logFC, log2 (fold change; 3D-cultured GMSCs over those 2D-cultured). **b** The upregulation of several Notch signaling genes in GMSCs cultured in 3D-collagen hydrogel was confirmed by quantitative RT-PCR as compared to those in 2D-cultured GMSCs. Data represent the mean ± SD, *n* = 3 biological replicates. *p < 0.05; **p < 0.01. Student’s two-tailed unpaired *t*-test (**b**). 2D or 2D-con, GMSCs cultured in 2D-conditions; 3D, GMSC cultured in 3D-collagen hydrogel.

**Fig. 6:**
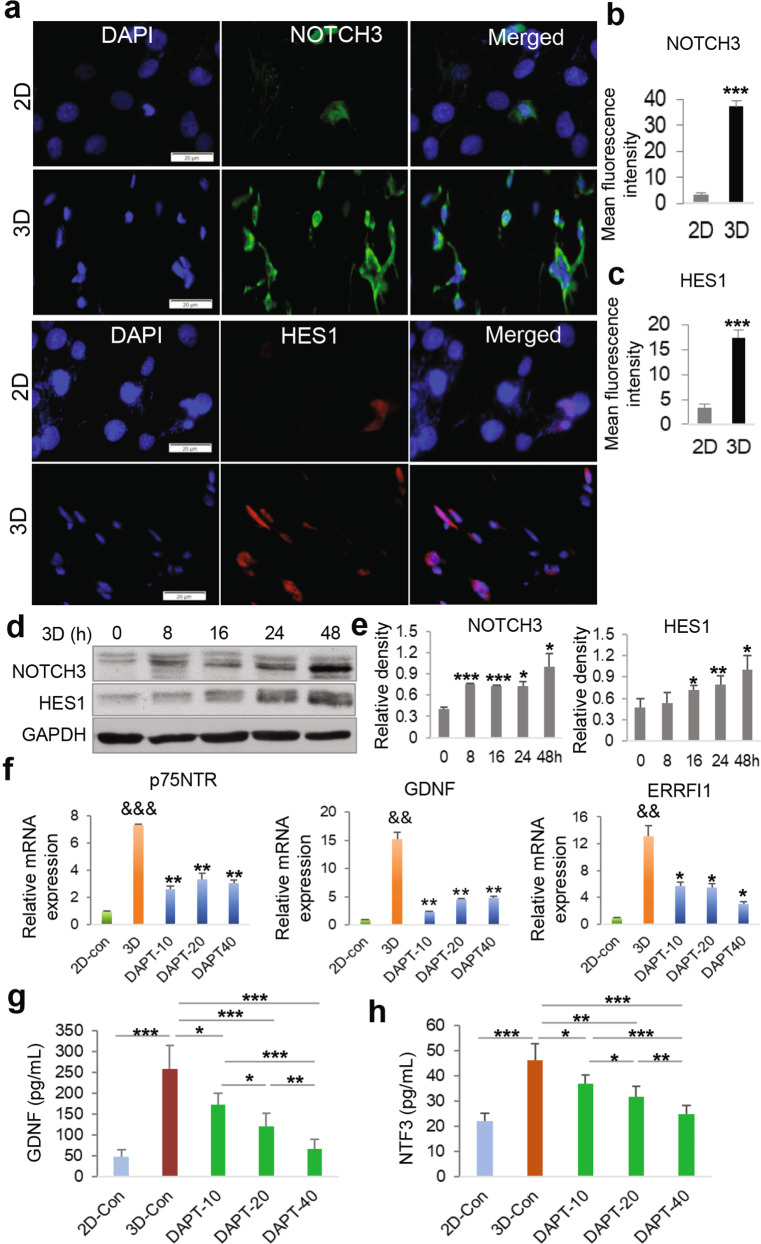
Blocking NOTCH signaling activation attenuated 3D collagen hydrogel-mediated upregulation of NCSC/SCP-related genes in GMSCs. GMSCs were encapsulated in 3D-collagen hydrogel (4 mg/mL) at a cell density of 2 × 10^6^/mL and cultured in complete α-MEM for 48 h. **a** Immunofluorescence (IF) staining for NOTCH3 (green color) and HES-1 (red color). Nuclei were counterstained with 4′, 6-diamidino-2-phenylindole (DAPI; blue). Scale bar = 20 µm. Quantification of IF intensity of NOTCH3 (**b**) and HES1 (**c**). **d** GMSCs encapsulated 3D collagen hydrogel were cultured for different time periods and the expression of NOTCH3 and HES1 proteins was determined by Western blot (WB). **e** Quantification of the relative WB densities of NOTCH3 and HES1 with GAPDH as the internal control. **f**−**h** GMSCs encapsulated in 3D-collagen hydrogel (4 mg/mL) at a cell density of 2 × 10^6^/mL were cultured in complete α-MEM in the presence of different concentrations of DAPT (10, 20, 40 µmol/L), a specific inhibitor of NOTCH activity, for 48 h. The mRNA expression of *p75*^NTR^, *Gdnf*, and *Errfi1* genes in 3D-cultured GMSCs was determined by qRT-PCR as compared to those in 2D-cultured GMSCs (**f**). *p < 0.05; **p < 0.01 (DAPT versus 3D control); ^*&&*^*p* < 0.01, ^&&&^*p* < 0.001 (3D versus 2D control). The secretion of GDNF (**g**) and NTF3 (**h**) in the supernatants were determined by ELISA. *p < 0.05; **p < 0.01; ***p < 0.001. Data represent the mean ± SD, *n* = 3 biological replicates. Student’s two-tailed unpaired *t*-test (**b**, **c**, **e**). One-way ANOVA with the Tukey’s post test (**f**−**h**). 2D or 2D-con, GMSCs cultured in 2D-conditions; 3D or 3D-Con, GMSC cultured in 3D-collagen hydrogel.

Due to the critical role of Notch signaling pathways in neural crest cell (NCC) fate determination and peripheral gliogenesis during development and differentiation of human pluripotent stem cells^38,43,44^, as well as in dedifferentiation of myelinating Schwann cells into a repair phenotype^45,46^, we then wonder whether Notch signaling was upregulated in GMSCs cultured in 3D-collagen hydrogel. Through DAVID Gene Functional Annotation/Classification of those significantly upregulated DEGs in 3D-cultured GMSCs, we identified 19 Notch signaling components, including Notch ligands (*DLL1*, *DLL4*, and *Jagged 2*), *Notch3* receptor, and canonical NOTCH-downstream transcription factors (*Hes1*, *Hes4*, *Hes7*, and *Hey1*) (Fig. 5a). The log2(FC) for *Dll1*, *Dll4*, *Jag2*, *Hes4*, and *Notch3* is ~2.5 for each gene, equals to ~five-fold change over those in 2D-GMSCs. The log2(FC) for *Hey1*, *Hes7*, and *Hes1* are 3.8, 4.8, and 6.1, respectively, which are equal to 13.8-, 27.3-, and 68.1-fold changes over 2D-cultued GMSCs, respectively (Fig. 5a). The upregulation of several major Notch signaling components at the mRNA level, e.g., *Notch3*, *Jag2*, *Dll1*, *Dll4*, *Hes1*, and *Hey1*, was further confirmed by qRT-PCR (Fig. 5b). Meanwhile, the increased expression of NOTCH3 and HES1 at the protein level was confirmed by IF staining (Fig. 6a–c) and Western blot (Fig. 6d, e), respectively. In addition, the presence of different concentrations of (2*S*)-*N*-[(3,5-Difluorophenyl) acetyl]-L-alanyl-2-phenyl] glycine 1,1-dimethylethyl ester (DAPT), a specific NOTCH inhibitor^38^, robustly abrogated the upregulated mRNA expression of *p75*^NTR^, *Gdnf*, *Errfi1* genes in GMSCs in cultured 3D-collagen hydrogel (Fig. 6f). Meanwhile, blocking NOTCH activity significantly abrogated the increased secretion of GDNF and NTF3 in GMSCs cultured in 3D-collagen hydrogel (Fig. 6g, h). These compelling results suggest that the activation of NOTCH signaling pathway may play an important role in 3D collagen hydrogel-driven conversion of GMSCs into NCSC/SCP-like cells.

### Harnessing 3D collagen hydrogel-directed conversion of GMSCs into NCSC/SCP-like cells to generate functionalized nerve guidance conduits

**Fig. 7:**
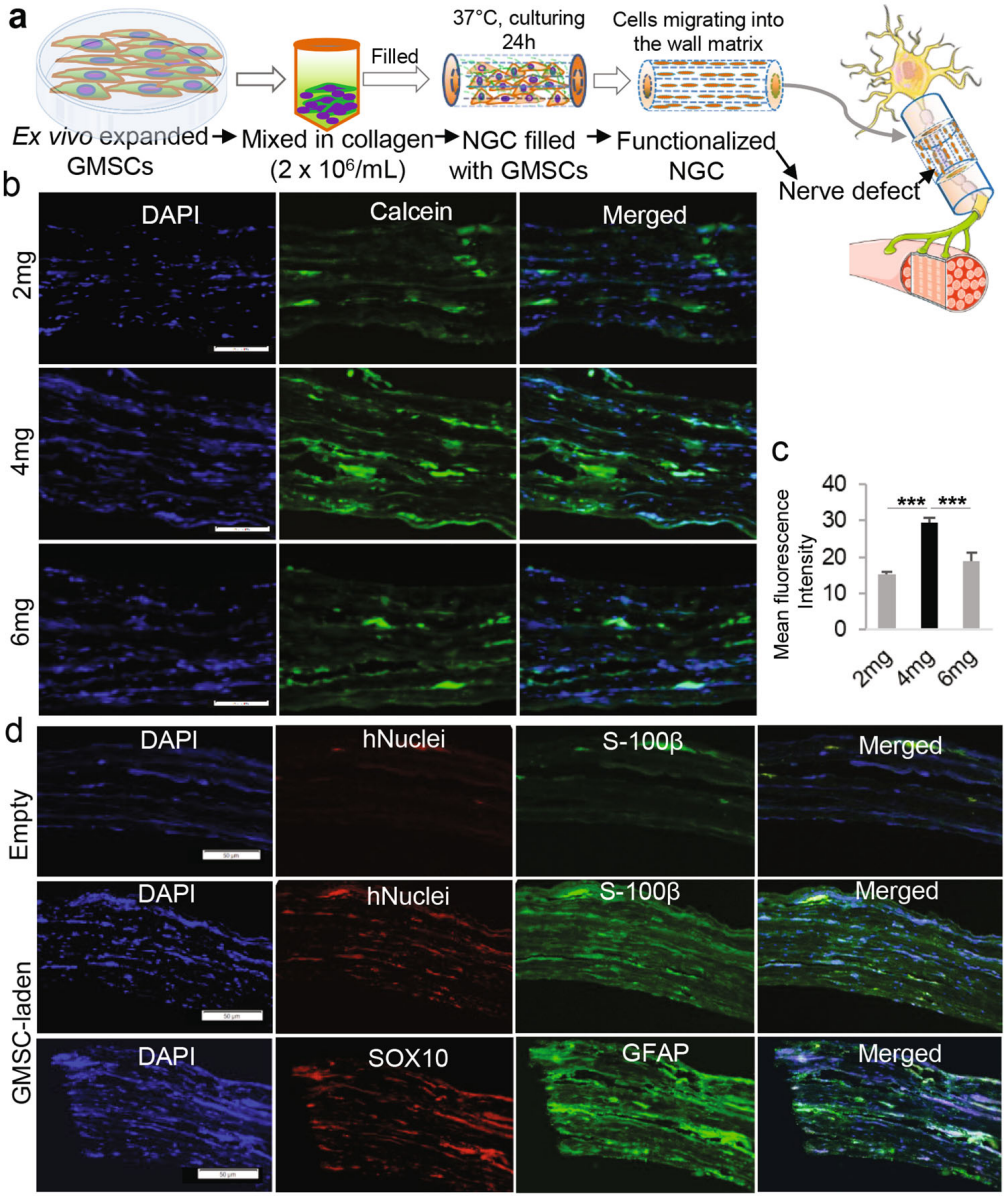
Generation of functionalized NGCs by harnessing 3D collagen hydrogel-directed conversion of GMSCs into NCSC/SCP-like cells. **a** GMSCs were encapsulated in 40 µl of 3D-collagen hydrogel at different concentrations (2, 4, 6 mg/mL) and a cell density of 2 × 10^6^/mL and then filled into AxoGuard Nerve protector or connector (NGC) (10 mm in length and 2 mm in inner diameter). Then, the constructs (NGC containing 3D collagen hydrogel encapsulated with GMSCs) were cultured for 24 h in complete α-MEM. Portions of this figure were made using templates from SMART SERVIER MEDICAL ART (https://smart.servier.com). **b** Before harvesting, the NGC constructs were labeled with 10 µM calcein-AM at 37 °C for 30 min. Cryosections were cut and the migrated cells labeled with calcein-AM (green color) in the wall matrix were observed under a fluorescence microscope. Nuclei were counterstained with 4′, 6-diamidino-2-phenylindole (DAPI; blue). **c** Quantification of IF intensity of calcein-AM. **d** Cryosections of NGCs containing cell-free collagen hydrogel (Empty) or GMSC-laden collagen hydrogel at a concentration of 4 mg/mL (Cell-laden) were prepared for dual-color immunostaining for human nuclei (hNuclei; red color), SOX10 (red color), S-100β (green color) or GFAP (green color). Nuclei were counterstained with 4′, 6-diamidino-2-phenylindole (DAPI; white or blue). Scale bar = 50 µm (**b**, **d**). ***p < 0.0001. Data represent the mean ± SD, *n* = 3 biological replicates. One-way ANOVA with the Tukey’s post test (**c**). NGC, nerve guidance conduit.

The neural crest stem/progenitor cells not only possess multipotent stem cell-like characteristics such as self-renewal but also have potent migratory capacity^16^. We wonder whether we could harness this unique property to automatically generate functionalized nerve conduits (NGC) laden with GMSC-derived NCSC/SCP-like cells (GiSCs) directed by 3D-collagen hydrogel. To this end, GMSCs encapsulated in 3D-collagen hydrogel with different concentrations (2, 4, 6 mg/mL) were filled into nerve connector/protector made of porcine small intestine submucosal extracellular matrix (SIS-ECM) and then cultured with regular MSC medium for 24 h (Fig. 7a). Calcein-AM staining indicated that GMSCs encapsulated in 3D-collagen hydrogel at 4 mg/mL exhibited the maximal transmigration into the wall matrix of the NGC as compared to the other two concentrations of hydrogel (Fig. 7b, c). The transmigration of GMSCs encapsulated in 3D-collagen hydrogel at 4 mg/mL into the wall matrix of NGCs was further confirmed by the positive expression of human nuclei (Fig. 7d). Meanwhile, GMSCs transmigrated into wall matrix of NGCs positively express glial/Schwann cell-related genes, such as S-100β, GFAP, and SOX10 (Fig. 7d), but also neurotrophic factors, GDNF and BDNF, but not NGF (Supplementary Fig. 5a−c). Taken together, these results demonstrated the feasibility of readily generating functionalized NGCs laden with GiSCs by harnessing the unique behavior and fate of GMSCs directed by 3D-collagen hydrogel.

### Implantation of functionalized nerve conduits laden with GiSCs facilitated functional recovery and axonal regeneration of transected rat facial nerves

**Fig. 8:**
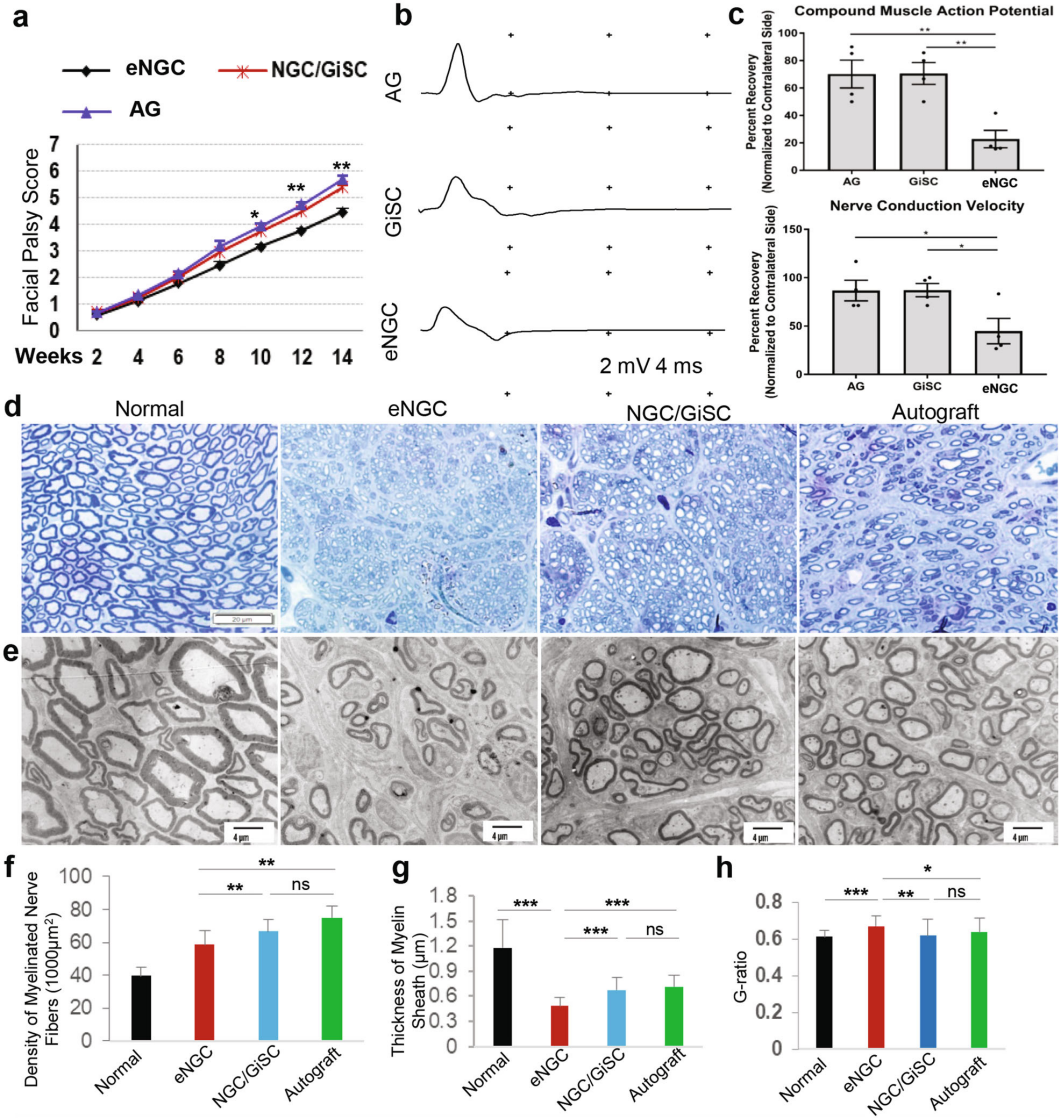
Implantation of NGC/GiSCs promotes functional recovery and axonal regeneration of transected rat facial nerves. **a** Longitudinal evaluation of facial palsy score in rats following implantation of empty nerve conduits (eNGC), nerve autograft (AG), or nerve conduits (NGC) laden with GiSCs for up to 14 weeks. Data represent the mean ± SD (*n* = 6 rats). *p < 0.05, **p < 0.01 (NGC/GiSC vs eNGC). Student’s two-tailed unpaired *t*-test (**a**). **b**, **c** Compound muscle action potential (CMAP) recordings of the vibrisal muscles of both the injury side and the contralateral normal side of rats following both proximal and distal stimulation. Motor nerve conduction velocity of both the injury side and the contralateral normal side of rats was calculated as described in “Methods”. Data represent the mean ± SD (*n* = 4 rats). *p < 0.05, **p < 0.01. One-way ANOVA with the Tukey’s post test (**c**). **d** Toluidine blue staining of semi-thin sections of the newly regenerated facial nerves from different groups of rats at 14 weeks post-injury and implantation. Scale bar = 20 µm. **e** Transmission electron microscopy (TEM) of ultrathin sections of the newly regenerated facial nerves from different groups of rats at 14 weeks post-injury and implantation. Scale bars, 4 µm. **f** Quantification of density of myelinated axons (the number of myelinated axons/1000 µm^2^). **g** Quantification of the thickness of the myelin sheaths. **h** Calculation of the G-ratios (the inner axonal diameter/the outer myelinated fiber diameter). Data represent the mean ± SD (*n* = 3 rats). *p < 0.05, **p < 0.01, ***p < 0.001; ns no significance. One-way ANOVA with the Tukey’s post test (**f**−**h**). AG autograft; eNGC empty nerve conduit; NGC nerve conduit; GiSC GMSC-derived NCSC/SCP-like cells.

We then evaluated the regenerative potentials of functionalized nerve conduits laden with GiSCs in a transected facial nerve defect model in rats. Clinically, longitudinal nerve function assessment indicated that both groups of animals implanted with nerve autografts and NGC/GiSCs showed significantly and comparably improved facial palsy scores as compared to those implanted with empty NGCs (Fig. 8a) (*p* < 0.01). At 14 weeks post-surgery, EMG analysis showed that implantation of nerve autografts and NGC/GiSCs exhibited comparable beneficial effects on the recovery of the compound muscle action potential (CMAP) of the vibrissal muscle and nerve conduction velocity (NCV), both of which were much more pronounced than those implanted with empty NGC (CMAP: AG or GiSC vs eNGC, *p* < 0.01; NCV: AG or GiSC vs eNGC, *p* < 0.05; Fig. 8b, c). Histologically, expression of Schwann cell and axonal markers, (S-100β and neurofilament, respectively) was significantly elevated in the graft site following repair with an autograft or NGC/GiSCs compared to empty NGC alone (Supplementary Fig. 6a−c). Toluidine blue staining and electron microscopy (EM) analyses showed that the newly regenerated facial nerves from animals implanted with either nerve autografts or NGC/GiSCs harbored well-organized nerve fibers, increased number of myelinated axons, and thicker myelin sheaths than those implanted with empty NGCs (Fig. 8d–h). Of note, even following implantation and surgery for 14 weeks, we noticed that GiSCs survived and were located on the periphery of newly regenerated nerves as identified by the positive immunostaining signals for human nuclei and GDNF (Supplementary Fig. 7). These findings suggest that functionalized nerve conduits laden with GiSCs significantly facilitate axonal regeneration and functional recovery of transected facial nerves of rats.

## Discussion

Neural crest stem cells (NCSCs) represent a unique population of multipotent stem cells formed at the neural plate border during vertebrate embryonic development and subsequently migrate to nearly all tissues of the body where they differentiate into a variety of specialized cells such as melanocytes, smooth muscle cells, peripheral glial/Schwann cells (SC), neurons, craniofacial soft and hard tissues^47^. Recently, cell lineage tracing studies have shown that a small population of NCSC-like cells exist throughout adulthood and may play an important role in tissue homeostasis and healing after injury^9,48^. Meanwhile, a population of mesenchymal stromal cells (MSCs) endowed with NCSC-like properties have been isolated and identified from various NC-derived tissues, such as dental pulp^6^, oral mucosa/gingiva^7–9,48,49^, bone marrow^10,11^, and skin epidermis^12^. Therefore, due to the wide existence and multipotency of adult tissue-derived NCSCs^14^, particularly those from the easily accessible orofacial tissues, they may represent an attractive source of supportive cells for cell-based regenerative therapy of various diseases, particularly, for nerve regeneration because of their intrinsic propensity to differentiate into glial cells and/or Schwann-like cells^15–17^. However, these adult tissue-derived NCSC-like cells tend to lose their neural stem cell properties and become more like heterogeneous MSCs following 2D-culture and expansion in vitro and then special conditions, e.g., crestosphere or neurosphere cultures, are required to maintain their NCSC properties^6–8,13^, potentially limiting novel tissue engineering and regenerative medicine approaches that require a sufficient number of NCSC-like cells for clinical translation.

Cell reprogramming is a process that reverts differentiated somatic cells into induced pluripotent stem cells (iPSCs), enabling the direct conversion from one cell lineage to another^27,28,50^. In tissue engineering and regenerative medicine, cell reprogramming provides a useful platform to generate a sufficient number of stem/progenitor and specialized cells with improved quality and functions for cell-based regenerative therapy^50^. Classically, cell reprogramming can be achieved through introducing a combination of defined transcription factors (TFs) or a single lineage-specifier TF and the use of a cocktail of small signaling molecules^20,24,26^. Recently, several lines of evidence have shown that somatic cells, e.g., fibroblasts^20–23^ and epidermal keratinocytes^24^, can be directly converted into NCSC/SCP-like cells via genetic introduction of a single/multiple transcriptional factors or non-genetic approaches with defined culture conditions, the use of a cocktail of small molecules and/or biomaterials/scaffolds with manipulated substrate stiffness^20,22,24–26^. GMSCs represent a unique subpopulation of adult stem cells of a neural crest-origin^49^. Recently, we have shown that ex vivo expanded human GMSCs could be directly converted into neural progenitor-like or NCSC-like cells through non-genetic approaches under defined culture conditions^42^. These findings support the notion that any strategy that starts with a cell type with the same developmental origin as the expected cell type imprinted with similar intrinsic gene regulatory networks, for example, converting adult NC tissue-derived cells to earlier precursors, e.g., NCSC-like cells, may represent a novel paradigm to achieve the efficient and reproducible outcome of cell phenotypic conversion.

In recent years, natural and synthetic biomaterial scaffolds have been explored as a novel paradigm to guide cell conversion or differentiation toward a specific cell lineage through changing their mechanical properties^27^. For instance, the use of soft hydrogel has been shown to facilitate iPSC generation through promoting mesenchymal-epithelial transition (MET) process^51^. When cultured in 3D scaffold with different matrix stiffness, human MSCs underwent either neurogenic differentiation from elastic modulus in 0.1−1kPa, or myogenic differentiation in 8−17kPa, or osteogenic differentiation in 25−40 kPa^28,52^. In the present study, we showed that when cultured in soft 3D methacrylated Type I bovine collagen hydrogel at 4 mg/mL, GMSCs of the NC-origin could be rapidly and consistently converted into NCSC/SCP-like state characterized by increased expression of a panel of NCSC/SCP-related genes and a simultaneous decrease in the expression of MSC-related genes. Our findings have demonstrated a novel approach for the efficient generation of NCSC/SCP-like cells by culturing GMSCs in 3D collagen hydrogel with an optimal stiffness (Figs. 1 and 2).

Several master signaling pathways, such as WNT, Sonic Hedgehog, bone morphogenetic proteins (BMPs), fibroblast growth factor (FGF), transformation growth factor (TGF)-β, and NOTCH signaling, have been identified as major nodes of the regulatory networks that control NC fate determination and different lineage specialization during embryonic development^14^ and induction of iPSCs toward NC fate^38^. Meanwhile, several lines of evidence have implicated the critical role of NOTCH signaling in governing the fate determination of NCSCs to glial cell lineages in the developing peripheral nervous system^43,44^ as well as in the conversion of myelinating Schwann cells into a repair phenotype^45,46^. In the present study, we identified a significant upregulation of a panel of Notch signaling components, particularly, DLL1, DLL4, JAG2, Notch3, Hes1, and Hey1, during 3D collagen hydrogel-directed conversion of NC-derived GMSCs into NCSC/SCP-like state (Figs. 5 and 6). These findings suggest that the intrinsic Notch signaling pathway imprinted in NC precursors and their derivatives could be harnessed to convert adult NC-derived cells, e.g., GMSCs, toward their precursor state.

To date, there is still lack of efficient therapies that ensure full regeneration and functional recovery of peripheral nerve injury (PNI) due to our limited understanding of the pathophysiology of PNI and mechanisms underlying nerve repair/regeneration. Currently, nerve autografts remain the gold standard for the treatment of injured nerves with a gap, but major shortcomings such as the limited availability, donor site morbidity, and the suboptimal clinical outcome, have significantly compromised their clinic use^53^. In the last two decades, much progress has been made in fabricating different types of nerve guide conduits (NGCs) as potential alternatives to nerve autografts, including the combinatory use of supportive cells and biological factors, in peripheral nerve repair/regeneration, but large variations exist in the clinical outcomes due to the differences in cell delivering strategies^33,34^. Herein, we found that GMSCs encapsulated in 3D-collagen hydrogel were converted into NCSC/SCP-like phenotype, which could spontaneously transmigrate into multilayered wall matrix of natural nerve conduits and express neurotrophic factors (Fig. 7; Supplementary Fig. 5). Moreover, implantation of functionalized nerve conduits laden with GiSCs significantly facilitated regeneration and functional recovery of transected facial nerves of rats (Fig. 8). These findings have demonstrated the feasibility to rapidly generate functionalized NGC by harnessing 3D collagen hydrogel-driven conversion of GMSCs into NCSC/SCP-like cells.

In conclusion, we have demonstrated that adult NC tissue-derived GMSCs encapsulated in 3D-collagen hydrogel could be rapidly converted into NCSC/SCP-like cells, which can spontaneously transmigrate and integrate into the wall matrix of natural nerve conduits made of porcine small intestine submucosal matrix (SIS-ECM), leading to rapid generation of functionalized NGCs with significantly improved therapeutic potentials in peripheral nerve repair/regeneration following implantation in vivo. Therefore, the present study has provided a platform for rapid, reproducible, and efficient fabrication of functionalized NGCs with translational potentials in clinic settings.

## Methods

### Animals

Female Sprague-Dawley rats aged 6−8 weeks old (weighing 200−250 g) were purchased from Charles River Laboratories. All animal procedures were approved by the Institutional Animal Care and Use Committee (IACUC) of University of Pennsylvania (Protocol No. 805451). Rats were group-housed in polycarbonate cages in the animal facilities with controlled temperature (23 ± 2 °C), 40–65% of humidity, and a 12 h light/dark cycle, fed with a standard laboratory diet and allowed *ad libitum* access to drinking water.

### Cell culture

Gingival tissues were obtained as remnants of discarded tissues from healthy human subjects aged from 20 to 40 years old, who underwent a dental procedure following informed consents. All procedures were approved by the Institutional Review Board (IRB) at University of Pennsylvania. Primary GMSCs were isolated, cultured and ex vivo expanded in complete alpha-minimum essential medium (α-MEM) supplemented with 1% L-glutamine, 10% FBS (Zen Bio), and 1% penicillin/streptomycin at 37 °C with 5% CO_2_^35^. Human bone marrow-derived mesenchymal stem cells (hBMSCs) were derived from bone marrow aspirations from healthy donors (Upenn Human Immunology Core) and cultured in complete alpha-minimum essential medium (α-MEM) supplemented with 1% L-glutamine, 10% FBS (Zen Bio), and 1% penicillin/streptomycin at 37 °C with 5% CO_2_. Cells less than six passages were used for experiments.

### Culture of GMSCs in 3D collagen hydrogel

According to our preliminary screening, we selected to use a purified methacrylated Type I bovine collagen (>98%) as the scaffold in the present study because of the following unique properties of this commercially available product from Advanced Biomatrix, Inc. (Carlsbad, CA): (1) The collagen is produced from telo-peptide intact bovine collagen and modified by reacting the free amines, primarily the ɛ-amines groups of the lysine residues as well as the α-amines groups on the N-termini, whereby approximately 40% of the total lysine residues of the collagen molecule have been methacrylate; (2) It can be easily prepared to form native-like 3D scaffolds with varying degree of stiffness by simply altering collagen concentrations^54^; (3) Collagen methacrylate is both thermoreversible and photo-crosslinkable^55^, being used as a rapidly self-assembling type I collagen to form cross-linked hydrogels for various tissue engineering applications, including 3D bioprinting^56^.

3D collagen hydrogel was prepared according to the manufacturer’s instructions. Briefly, 100 mg of the lyophilized methacrylated type I bovine collagen was dissolved in 16.7 mL of 20 mM acetic acid and mixed on a shaker at 2−10 °C until fully solubilized to make a stock gel solution at a concentration of 6 mg/mL. Then, the required volume of chilled neutralization solution (NS) was added to the calculated volume of the chilled collagen stock solution (85 µl NS: 1 mL collagen) and mixed quickly and thoroughly by pipetting. Afterwards, GMSCs resuspended in a calculated volume of chilled PBS were added into the collagen mixture and mixed quickly and thoroughly by pipetting to achieve a final collagen concentration at 4 mg/mL and a cell density at 2 × 10^6^/mL. Then, the collagen mixture encapsulated with GMSCs was dispensed in the desired culture plates and incubated at 37 °C for 20 min for gel formation followed by culturing in a complete α-MEM medium for different time periods. Afterwards, the constructs were harvested and cryosections were prepared for further immunofluorescence studies. Under certain conditions, the 2D plastic four-well chambered cell culture slides (Nunc^®^ Lab-Tek^®^ Chamber Slide^™^ system; Cat. #: C6932; Sigma) were pre-coated with 4 mg/mL methacrylated collagen hydrogel. Then, GMSCs were seeded on the top surface of the solidified hydrogel and cultured under the same condition for 48 h. To recover cells from 3D-collagen hydrogels, the cell-laden scaffolds following culture for 48 h were digested with collagenase I (2 mg/mL) at 37 °C on a shaker for 30 min. Single cells were collected for further analysis.

### Osteogenic differentiation

GMSCs from 2D-cultures or recovered from 3D-collagen hydrogel were plated at 5 × 10^5^ cells/well in six-well plates in MSC growth medium, allowed to adhere overnight, and replaced with osteogenic induction medium supplemented with dexamethasone, l-glutamine, ascorbic acid, and β-glycerophosphate. 4–5 weeks later, the in vitro mineralization was assayed by Alizarin Red S (Sigma-Aldrich) staining and quantified by an acetic acid extraction method^35^.

### Adipogenic differentiation

GMSCs from 2D-cultures or recovered from 3D-collagen hydrogel were plated at 5 × 10^5^ cells/well in six-well plates in MSC growth medium, allowed to adhere overnight, and replaced with adipogenic induction medium supplemented with 10 μM human insulin, 1 μM dexamethasone, 200 μM indomethacin, and 0.5 mM 3-isobutyl-1-methylxanthine (Sigma-Aldrich). Two weeks later, intracellular lipid vacuoles characteristic of adipocytes were determined by Oil Red O staining and the dye content was quantified by isopropanol method^35^.

### Calcein-AM staining

NGCs filled with GMSC-laden 3D collagen gels (2, 4, 6 mg/ml at a cell density of 2 × 10^6^/mL) were cultured in complete α-MEM for 24 h. Before harvesting, Calcein-AM (Cat. # 564061; BD Pharmingen) was added into the culture at a final concentration of 1 µM and incubated with at 37 °C for 30 min. Cryosections of the NGC constructs were cut and the signal of Calcein-AM staining was observed under a fluorescence microscope.

### RNA extraction, library construction, and RNA-Seq

RNA was extracted from the samples according to the instruction manual of the TRIzol reagent (Invitrogen, Carlsbad, CA). RNA concentration and purity were measured using a NanoDrop 2000 Spectrophotometer. RNA integrity was assessed using the RNA Nano 6000 Assay Kit of the Agilent Bioanalyzer 2100 system (Agilent Technologies, CA, USA). High-quality RNA was sent to LongseeMed Corporation (Guangzhou, China) for cDNA libraries construction and sequencing run on the Illumina Xten. mRNA was purified by the interaction of the poly (A) tails and magnetic oligo (dT) beads. RNA sequencing libraries were generated using the NEBNext, Ultra RNA Library Prep Kit for Illumina (New England Biolabs, Ipswich, MA, U.S.A.) with multiplexing primers, according to the manufacturer protocol. The cDNA library was constructed with average inserts of 300 bp (250−300 bp), with non-stranded library preparation. The cDNA was purified using AMPure XP Beads (Beckman Coulter, Inc.). The short cDNA fragments were subjected to end repair, adapter ligation. Then, the suitable fragments were selected by Agen court AMPure XP beads (Beckman Coulter, Inc.) and enriched by PCR amplification.

### Data analysis for RNA-seq

Base quality value and base distribution of raw data were detected to control the quality of initial RNA-seq data by Fastp. In the trimming process, the sequencing adapters, 3 leading and 3 trailing bases were first trimmed. The reads were then scanned from both ends, using a 4bp-wide sliding window, within which the low quality (lower than Q30) bases were trimmed. Finally, the resulting reads of length at least 50 bases were selected for further analysis. The read alignment is done using HISAT2 v.2.0.5 software with Ensembl GRCh38 genome as reference genome. Transcripts Assembly is done using StringTie v.1.3.3b software. Significant DE genes or transcripts (*q*-value < 0.05) were extracted by edgeR (R package) for each comparison groups. Differentially expressed genes (DEGs) were analyzed with gene ontology enrichment analysis and KEGG by R software with cluster Profiler package. Significant GO or KEGG terms (FDR-value < 0.05) were extracted using hypergeometric distribution. DAVID Gene Functional Classification Tool (https://david.ncifcrf.gov/) was employed to identify the biological functions of the genes related to NCSCs and Notch signaling pathways.

### Quantitative real-time polymerase chain reaction (qRT-PCR)

Total RNA was extracted using the Trizol reagent (Invitrogen) and RNA concentration and purity were measured using a NanoDrop 2000 Spectrophotometer. The first-strand cDNA was synthesized using the High-Capacity cDNA Reverse Transcription Kits (Applied Biosystems). The quantitative real-time PCR (qRT-PCR) was performed using cDNA as the template in a 20 μl reaction mixture containing FastStart SYBR Green Master (Qiagen), and a specific pair of primers of each cDNA on Bio-Rad CFX96 Touch Real-Time PCR Detection System. The amplification steps included denaturation at 95 °C for 15 min, followed by 40 cycles of denaturation at 94 °C for 15 s, annealing at 55 °C for 30 s, and extension at 72 °C for 30 s. Data were obtained from three independent samples. The relative gene expression was quantified using the delta−delta Ct method (^ΔΔ^CT) with the expression of *Gapdh* as an internal control. The relative fold-change in a specific gene of interest from the control was calculated using the 2^−∆∆ct^ method. The sequences of the primers are listed in Supplementary Table 1.

### Natural nerve conduits laden with Schwann-like cells reprogrammed from GMSCs in 3D collagen hydrogel

Collagen hydrogel (40 µl at a final concentration of 4 mg/mL) encapsulated with GMSCs (2 × 10^6^/mL) was filled into the commercially available AxoGuard Nerve protector or connector (2 mm in internal diameter × 10 mm length) made of porcine submucosal ECM (Cook Biotech) and incubated at 37 °C for 20 min for gel formation followed by culturing in complete α-MEM medium for 24 h. Afterwards, the constructs were used for further in vivo studies.

### Immunofluorescence studies

Cryosections prepared from 3D-collagen gel or GMSC-seeded nerve conduits were blocked and permeabilized for 1 h at room temperature in PBS with 2.5% goat serum and 0.5%Triton X‐100, followed by incubation with the following primary antibodies at the appropriate dilution overnight at 4 °C: p75 (mouse IgG, 1:200, Sigma), SOX-9 (rabbit IgG, 1:200, Cell Signal Tech), SOX-10 (mouse IgG, 1:200, R & D), S-100β (rabbit IgG, 1:200, Boster Biological Tech), NOTCH3 (rabbit IgG, 1:200, Abcam), HES1 (rabbit IgG, 1:200, Cell Signaling Tech), vinculin (mouse IgG, 1:400, Millipore), TRITC-conjugated phalloidin (1:400, Millipore), BDNF (rabbit IgG, 1:200, Abcam), GDNF (rabbit IgG, 1:200, Abcam), or NGF (rabbit IgG, 1:200, Abcam). After washing with PBS, cells were incubated with appropriate secondary antibodies at room temperature for 1 h: goat anti-rabbit IgG–AlexaFluo-488 (1:300, BioLegend). Isotype-matched control antibodies (BioLegend) were used as negative controls. Nuclei were counterstained with 4′, 6-diamidino-2-phenylindole (DAPI). Images were captured using Olympus inverted fluorescence microscope (IX73). For semi-quantitative analysis, at least six randomly selected regions of interesting (ROI) were visualized and the integrated immunofluorescence intensity was measured using Olypus cellSens^TM^ imaging software.

### Western blot

Cells cultured in the 3D-collagen gel were recovered following enzymatic dissociation with collagenase 1 (2 mg/mL) and whole cell lysates were prepared by incubation with radioimmunoprecipitation (RIPA) assay buffer (Santa Cruz) supplemented with a cocktail of protease inhibitors (Santa Cruz) and the total protein concentrations were determined using bicinchoninic acid (BCA) method (BioVision). Then 30 µg of proteins were subjected to SDS-polyacrylamide gel electrophoresis before being electroblotted onto a 0.2 μm nitrocellulose membrane (GE Healthcare). After blocking with 5% nonfat dry milk in TBST [25 mmol/L Tris (pH, 7.4), 137 mmol/L NaCl, 0.5% Tween20], membranes were incubated at 4 °C overnight with following primary antibodies: p75 (1:1000, Cell Signaling), NOCTH3 (1:1000, Abcam), HES1(1:1000, Cell Signaling). GAPDH (1;2000, Cell Signaling) was used as a loading control. After extensively washing, membranes were incubated with horseradish peroxidase (HRP)-conjugated secondary antibodies (Santa Cruz), and blot signals were developed with ECL^TM^ Western Blotting Detect Reagents (GE Health Care). All blots were derived from the same experiment and processed in parallel. Uncropped Western blotting images were provided in Supplementary Fig. 8 with the size markers labeled.

### Flow cytometry

2D-cultured GMSCs or GMSCs recovered from 3D-collagen gels via digestion with collagenase I was immunostained with specific antibodies for human CD90, CD44, CD73 (1:200, BioLegend) or p75(1:200, Sigma) or an isotype control, followed by incubation with Alexa Fluor 488-conjugated secondary antibodies. The cell samples were analyzed by BD FACSCalibur Flow Cytometer. Data were processed and analyzed by FlowJo software.

### Surgical procedures of facial nerve transection

Transected facial nerve defects were created in adult Sprague-Dawley rats as we described recently^42,57^. Briefly, a 6-mm gap was made in buccal branch of the facial nerve, and the proximal and distal stumps were bridged by an 8 mm long nerve autograft, empty NGC or NGC laden with GMSC-derived NCSC/SCP-like cells (NGC/GiSC). Two 8-0 Ethilon interrupted sutures were applied at each side of the gap to stabilize the grafts. To block the signal to the whisker pad, a 6-mm defect was created in the marginal mandibular branch and ligated with 8/0 Ethilon interrupted sutures.

### Facial functional analysis using the facial palsy score

Facial palsy scores were blindly evaluated from animals in different treatment groups every week until the termination of the study according to the standards as described previously^42,57^. The facial palsy score was valued based on the following functional evaluation: (1) symmetry of the vibrissae at rest (0, asymmetry; 0.5, slightly; 1, normal); (2) motion of the vibrissae (0, no motion; 1, minor trembling; 2, effective movement; 3, normal); (3) symmetry of the nose at rest (0, asymmetry; 0.5, slightly; 1, normal); (4) motion of the nose (0, asymmetry; 1, slightly; 2, normal). A maximum seven-point indicates a normal midface without facial palsy, while a zero-point indicate complete facial palsy of the midface.

### Electrophysiological analysis

Electrophysiological analysis was performed at 14-weeks post transection of facial nerves of rats^42,57,58^. At the terminal time point, the nerve was transcutaneously stimulated using a monopolar stimulating electrode positioned proximal or distal to the repair site. CMAP recordings were obtained following stimulation from the active monopolar electrode placed in the muscle belly of the vibrisal muscles and reference electrode in the corresponding tendon. A train of five pulses was averaged to reduce background noise. The peak-to-baseline CMAP amplitude and latency were measured, and the nerve conduction velocity (NCV) was calculated using the onset latency and distance relative to the recording electrode for the two stimulation sites. CMAP latency was measured as the initial depolarization from the baseline after the stimulus artifact. To calculate percent recovery, CMAP values were normalized to the contralateral side.

### Immunohistochemical studies

The facial nerves were harvested 14 weeks post-injury and implantation of nerve conduits. The tissue samples were fixed in 4% PFA for 24 h and cryoprotected in 10, 20, and 30% sucrose and embedded in O.C.T. and 10 µm-thick cryostat sections were cut. After blocking and permeabilization in PBS containing 2.5% goat serum and 0.5% Triton X‐100 at room temperature for 1 h, the sections were incubated with primary antibodies for S-100β (1:200) and neurofilament (1:200) overnight at 4 °C, followed by incubation with fluorescein-conjugated secondary antibodies for 1 h at room temperature. Isotype-matched control antibodies (BioLegend) were used as negative controls. Nuclei were counterstained with DAPI. The images were captured under a fluorescence microscope and the integrated immunofluorescence intensity for both NFL and S-100β in six randomly selected regions of interesting (ROI) was quantified using Olypus cellSens^TM^ imaging software.

### Morphological evaluation of rat facial nerves

The facial nerves were isolated and fixed with 2.5% glutaraldehyde overnight at 4 °C, and then post-fixed with 1% osmium tetroxide (OsO4) for 2 h, dehydrated, and embedded in epoxy resin. Semi-thin sections (1 µm) were cut vertically with an ultramicrotome (EM UC7i, Leica Microsystems, Denver, CO, http://www.leica-microsystems.com), stained with 1% toluidine blue solution, and examined under a light microscope (Olympus IX-73). The density of the myelinated fibers (fibers/1000 µm^2^) was analyzed from six non-overlapping visual fields per specimen. Ultra-thin sections (60 nm) were stained with lead citrate and uranyl acetate, and then examined under a transmission electron microscope (TEM; JEM-1400; JEOL, Tokyo, Japan, http://www.jeol.co.jp). The diameter of myelinated fibers, axons, and the thickness of the myelin sheath were evaluated by cellSens Dimension software (Olympus) and the G-ratio was calculated as the ratio of the inner axonal diameter to the total outer diameter of the fiber.

### Statistical analysis

Quantifications were performed from at least three independent experiments (biological replicates or donors) for cell studies in vitro and from three to six animals for in vivo experiments. Unpaired two-tailed Student’s *t*-test and one-way analysis of variance (ANOVA) with the Tukey’s post test were performed for pairwise and multi-group comparisons, respectively. Data were represented as mean ± SD and a *P* value of less than 0.05 (*p* < 0.05) was considered statistically significant. All analyses were performed with Excel data analysis or SPSS Statistics version 18.0 (IBM, Inc., Armonk, NY, USA).

### Reporting summary

Further information on research design is available in the Nature Research Reporting Summary linked to this article.

## Supplementary information

Supplementary Information

Reporting Summary

## Data Availability

The data that support the findings of this study are available from the corresponding author upon reasonable request. The raw and processed RNA-seq were deposited in the NCBI Gene Expression Omnibus (GEO) public database repository (accession number GSE180519).
