# Supplementary material for: Harnessing 3D collagen hydrogel-directed conversion of human GMSCs into SCP-like cells to generate functionalized nerve conduits
Source: NPJ Regen Med. 2021 Sep 30;6:59. doi: 10.1038/s41536-021-00170-y (PMC8484485; doi:10.1038/s41536-021-00170-y)
Supplement: Supplementary file 1 — Supplementary Information [file 41536_2021_170_MOESM1_ESM.pdf]

Harnessing 3D collagen hydrogel-directed conversion of human GMSCs into SCP-like cells to generate functionalized nerve conduits

Qunzhou Zhang, Phuong Nguyen, Justin C. Burrell, Jincheng Zeng, Shihong Shi, Rabie M. Shanti, Grace Kulischak, D. Kacy Cullen, Anh D. Le

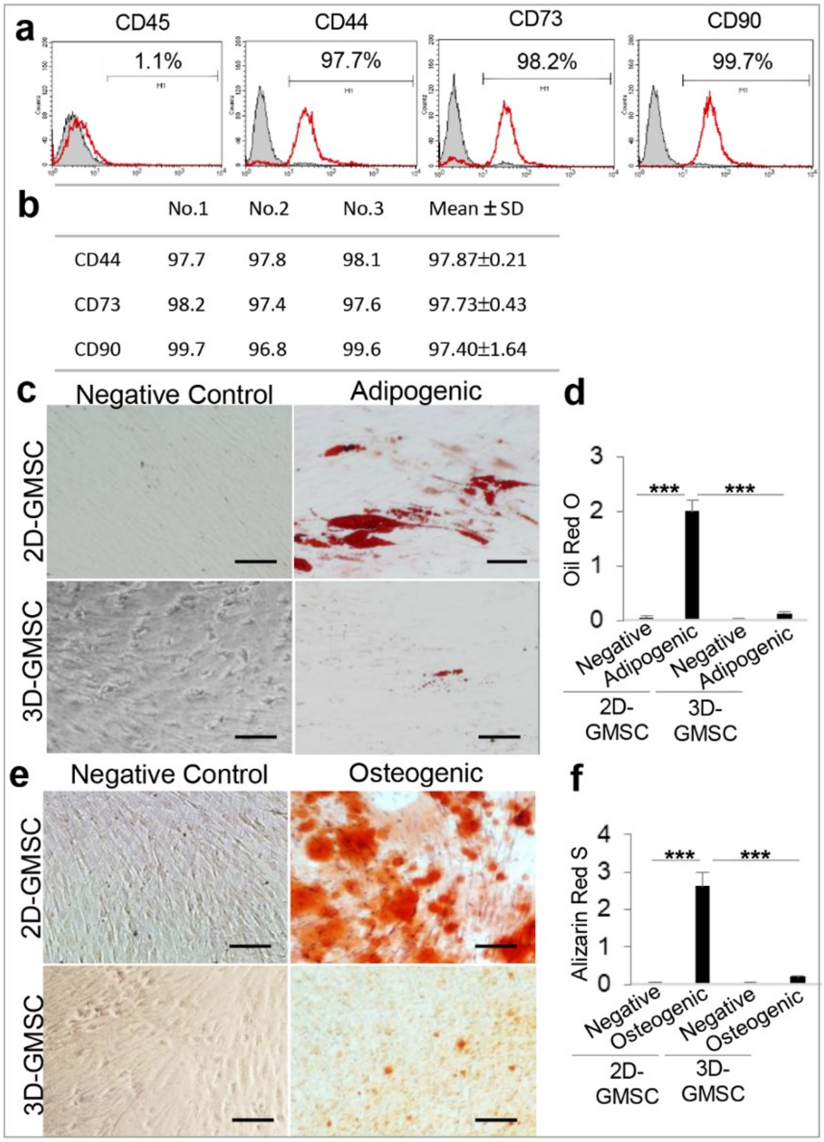

Supplemental Fig. 1. Characterization of gingival derived mesenchymal stem cells (GMSCs). **a** Representative histograms of flow cytometric analysis of mesenchymal stem cell-related cell surface markers, CD44, CCD73, and CD90, in GMSCs. **b** Expression of MSC-related cell surface markers in GMSCs derived from three healthy donors as determined by flow cytometry. **c** Adipogenic differentiation of GMSCs cultured under 2D cultures or recovered following culturing in 3D-colllagen hydrogel for 48h. Adipocytes were determined by Oil Red O staining. **d** Quantification of Oil Red O contents. **e** Osteogenic differentiation of GMSCs cultured under 2D cultures or recovered following culturing in 3D-colllagen hydrogel for 48h. Osteocytes were determined by Alizarin Red S staining. **f** Quantification of Alizarin Red S contents. Scale bar=50μm (**c**, **e**). Data represent the mean ± SD, n=3 biological replicates. ***p<0.001. Student's two-tailed unpaired t-test (**d**, **f**). 2D, GMSCs cultured in 2D-conditions; 3D, GMSC cultured in 3D-collagen hydrogel.

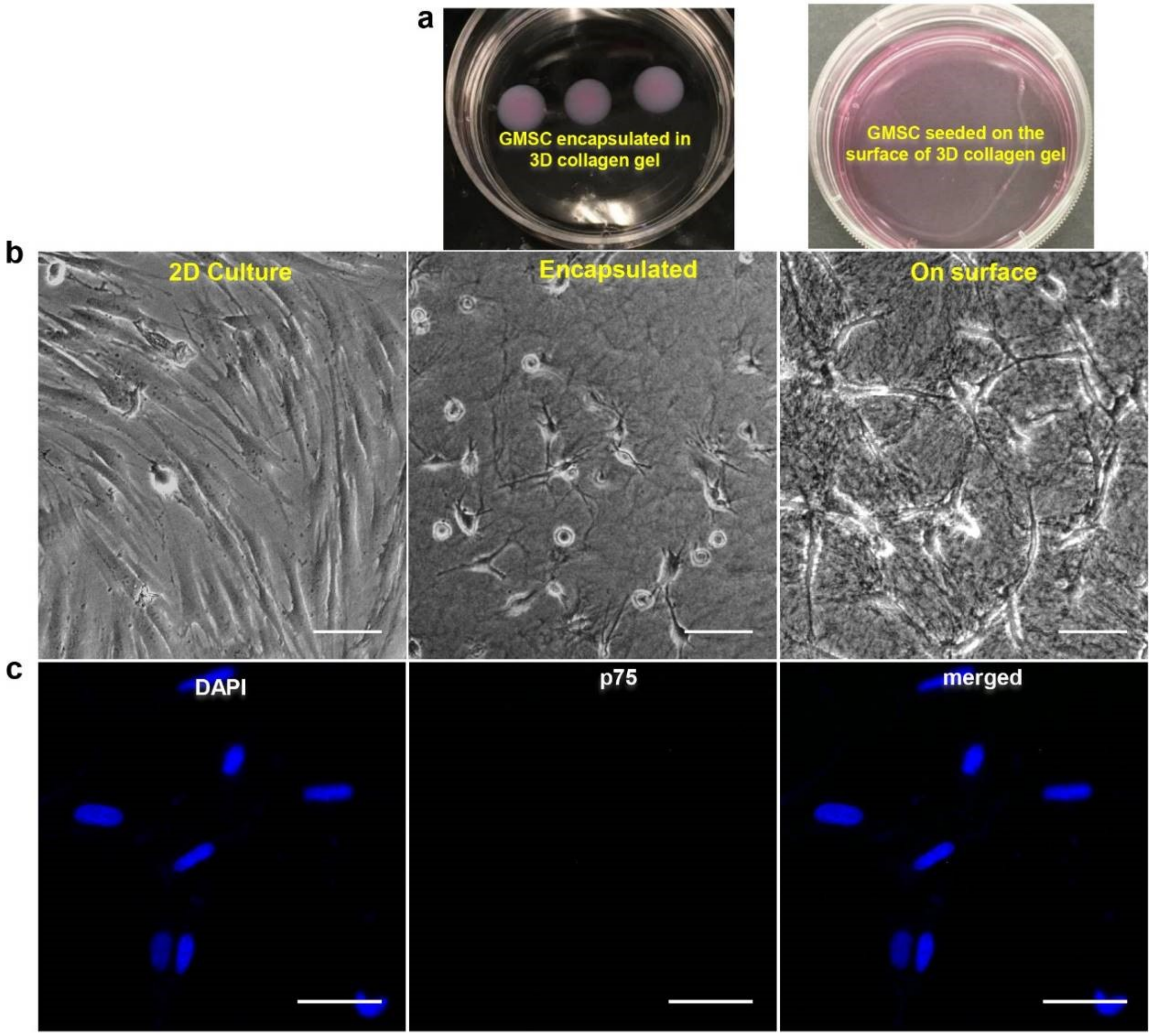

Supplemental Fig. 2. GMSCs cultured on the top surface of the methacrylated 3D-collagen hydrogel. a The 2D plastic 4-well chambered cell culture slides were pre-coated with 4mg/mL methacrylated collagen hydrogel. GMSCs then were seeded on the top surface of the solidified hydrogel and cultured in complete α-MEM for 48h. **b** The cellular morphology of GMSCs cultured under 2D plastic culture, encapsulation in 3D-collagen hydrogel, or on the top surface of the solidified hydrogel, respectively. **c** Immunofluorescence staining showed no increase in the expression of $p75^{NTR}$ in GMSCs cultured on the top surface of the solidified collagen hydrogel. Scale bar=50μm (**b**, **c**). Images are representative of three independent experiments (biological replicates).

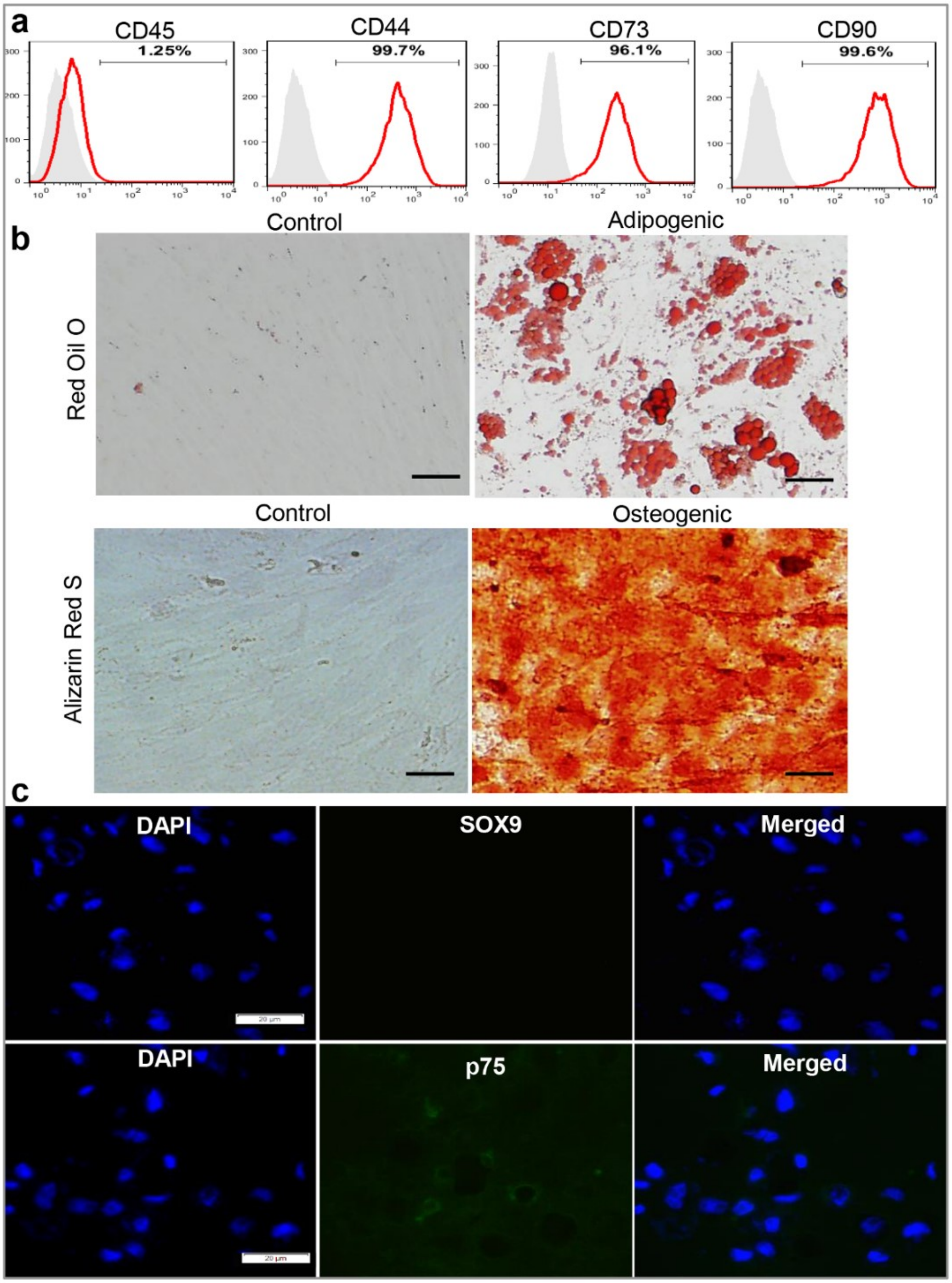

Supplemental Fig. 3. BMSCs cultured in methacrylated 3D-collagen hydrogel. a Flow cytometric analysis of MSC-associated cell surface markers on human bone marrow-derived mesenchymal stem cells (hBMSCs). **b** Adipogenic and osteogenic differentiation of hBMSCs as determined by Red Oil O and Alizarin Red S staining, respectively. Scale bar=50µm. **c** hBMSCs were encapsulated in 3D-collagen hydrogel (4mg/mL) at a cell density of 2×10^6/mL and cultured in complete α-MEM for 48h. Cryosections of the 3D-collagen gels laden with hBMSCs were immunostained with a specific antibody for SOX9 or $p75^{NTR}$ followed by incubation with Alexa Fluor 488-conjugated secondary antibody. Nuclei were counterstained with 4’, 6-diamidino-2-phenylindole (DAPI; white or blue). Scale bar=20µm. Data are representative of three independent experiments (biological replicates).

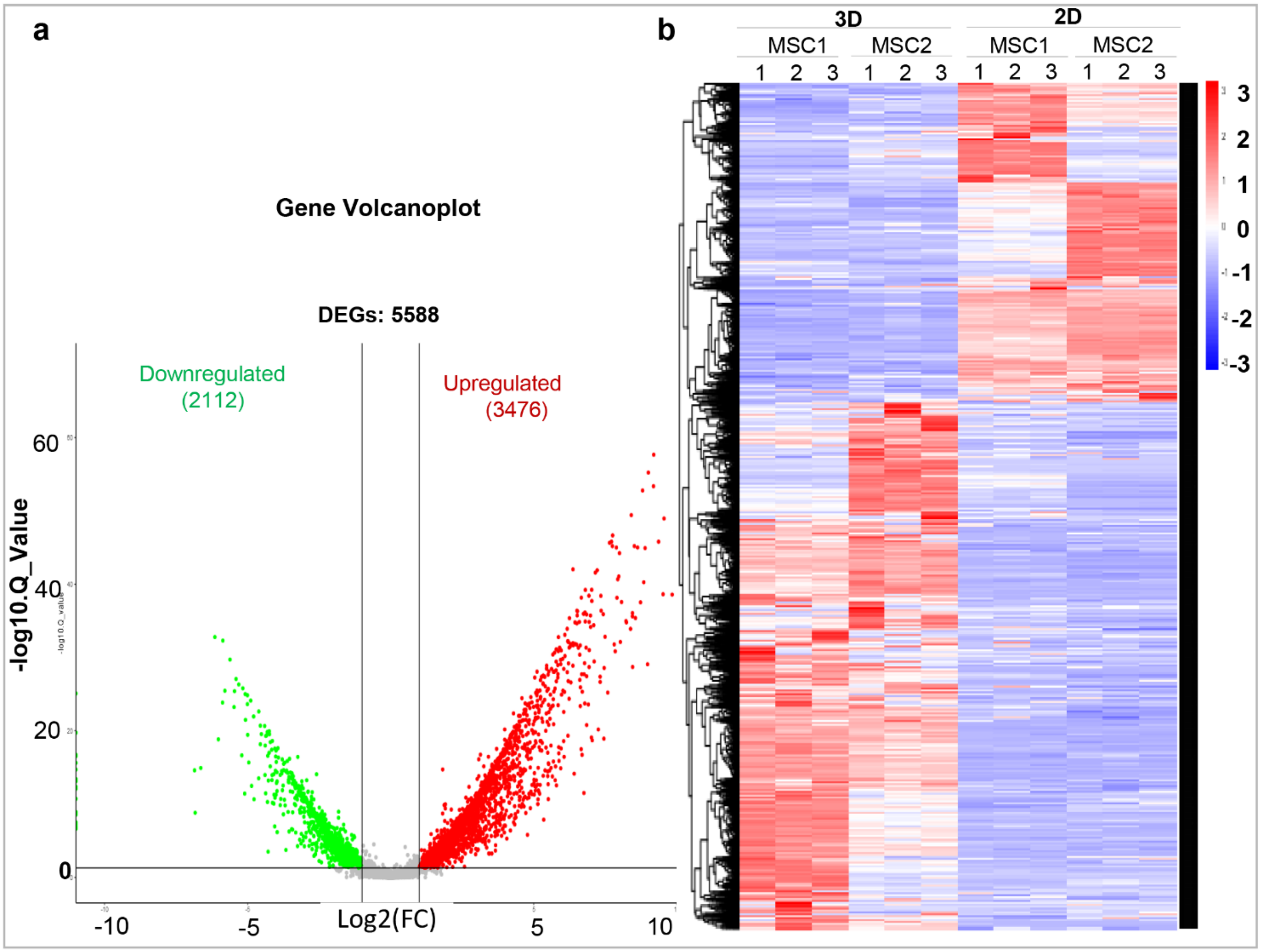

Supplemental Fig. 4. Gene expression profiling by *RNA-seq*. GMSCs were cultured under 2D- and 3D-collagen hydrogel conditions for 48h, respectively, and total RNA was extracted for RNA-seq. **a** Volcanoplot showing a total of 5588 differentially expressed genes (DEGs) with a fold change (FC) ≥1 (3D-cultured GMSCs *versus* vs 2D-cultured counterparts). **b** Heatmap showing relative representation of significant differentially expressed genes (DEGs) clustered according to expression pattern across samples (two biological replicates, GMSC1 and GMSC2; three technique replicates, 1, 2, 3). Red represents high expression and blue represent low expression. DEGs, differentially expressed genes; 2D, GMSCs cultured in 2D-conditions; 3D, GMSC cultured in 3D-collagen hydrogel.

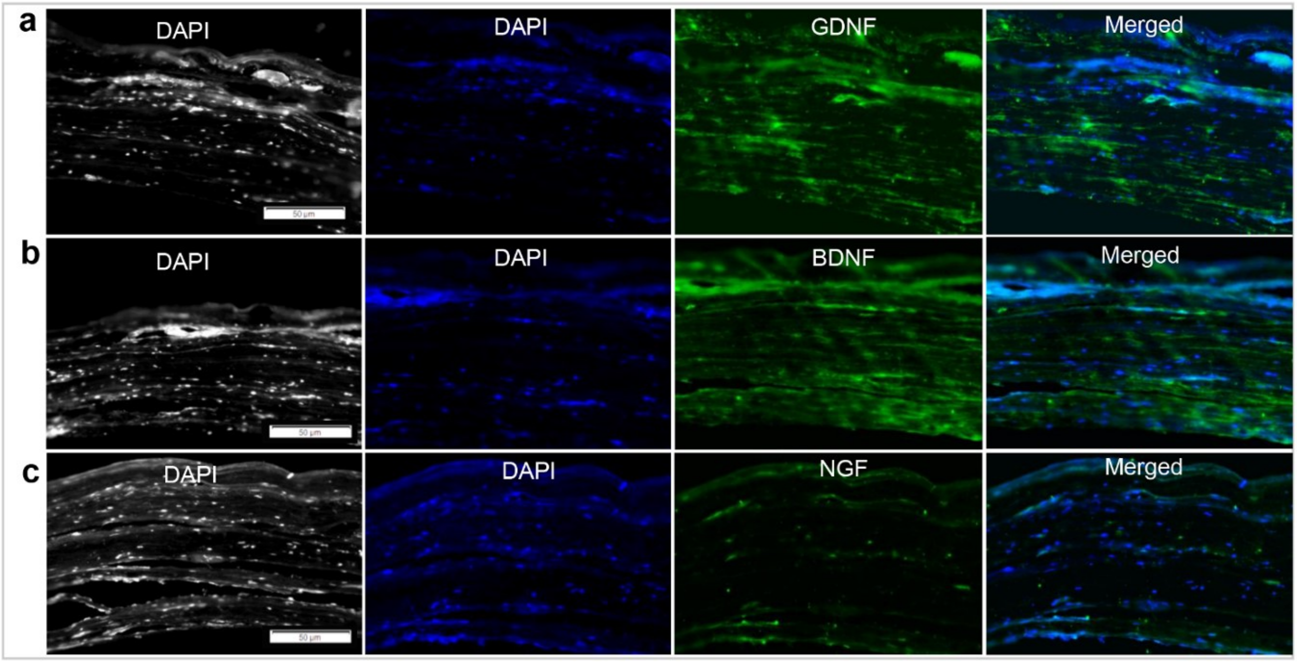

Supplemental Fig. 5. GMSC-derived NCSC/SCP-like cells encapsulated in 3D-collagen hydrogel transmigrated into the wall matrix of nerve conduits and expressed neurotrophic factors. GMSCs were encapsulated in 3D-collagen hydrogel at a final concentration of 4mg/mL and a cell density of 2×10^6/mL and filled into AxoGuard Nerve protector or connector (10mm in length and 2mm in inner diameter). Then, the constructs (nerve conduits containing 3D collagen hydrogel encapsulated with GMSCs) were cultured for 24h in complete α-MEM for 24h. Cryosections of nerve conduits were prepared for immunostaining with specific antibodies for GDNF (**a**), BDNF (**b**), or NGF (**c**), followed by incubation with Alexa Fluor 488-conjugated secondary antibodies. Nuclei were counterstained with 4', 6-diamidino-2-phenylindole (DAPI; white or blue). Scale bars=50µm. Images are representative of three independent experiments (biological replicates).

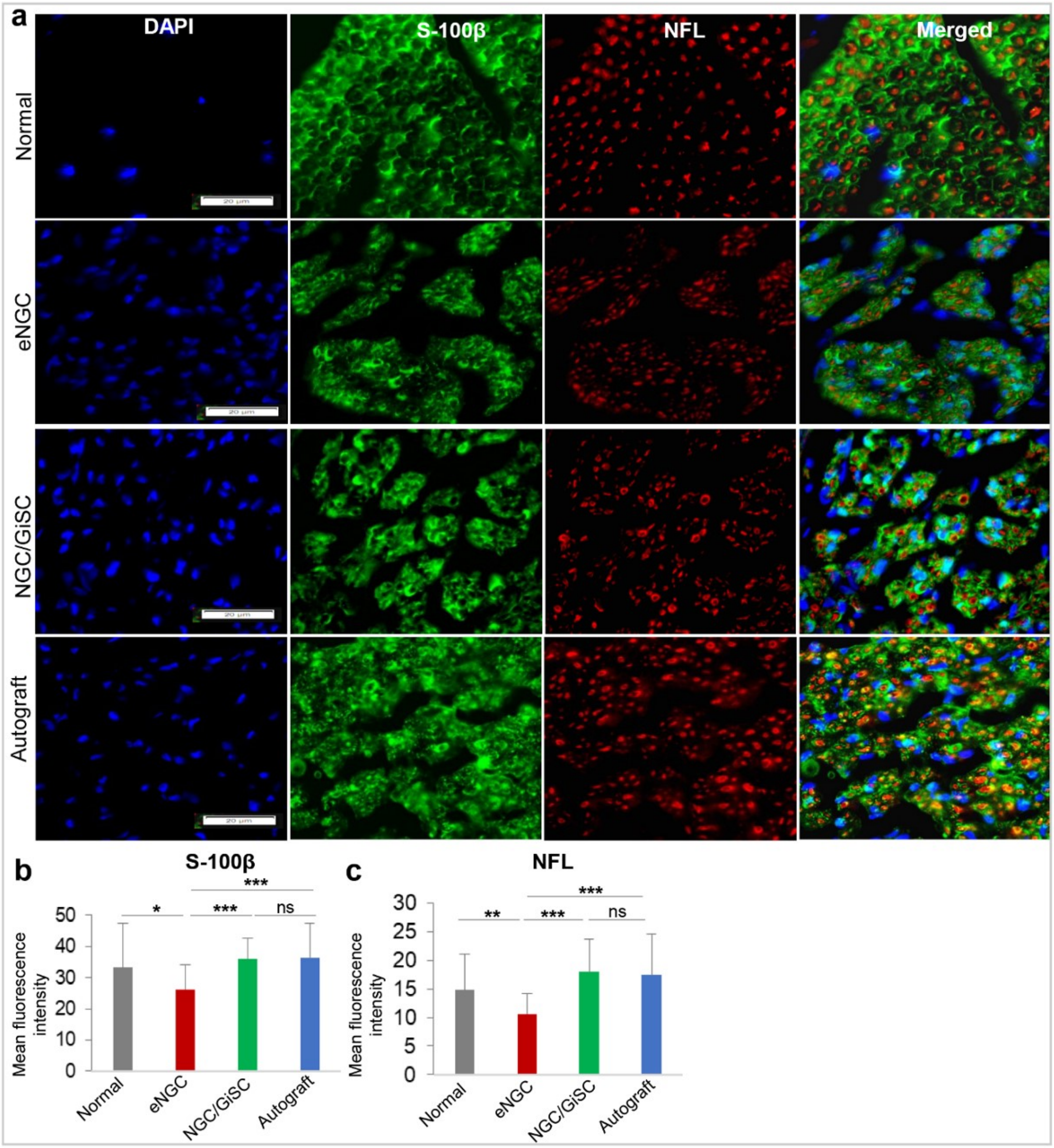

Supplemental Fig. 6. Implantation of NGC/GiSCs promotes axonal regeneration of transected facial nerves of rats. a At 14 weeks post-injury and implantation, the newly regenerated facial nerves were harvested and cross-sectional cryosections (10µm in thickness) were cut for immunofluorescence (IF) studies on the protein expression of S-100β and neurofilament (NFL). Nuclei were counterstained with 4', 6-diamidino-2-phenylindole (DAPI; blue). Scale bars=50 µm. **b, c** Quantification of the IF intensity of S-100β and neurofilament (NFL) expressions of 6 randomly selected regions of interesting (ROIs). Data represent the mean ± SD, n=3 rats. *p<0.05, **p<0.01, ***p<0.001; ns, no significance. One-way ANOVA with the Tukey's post test (**b**, **c**). NGC, nerve guidance conduit; eNGC, empty nerve conduit; GiSCs, GMSC-derived NCSC/SCP-like cells.

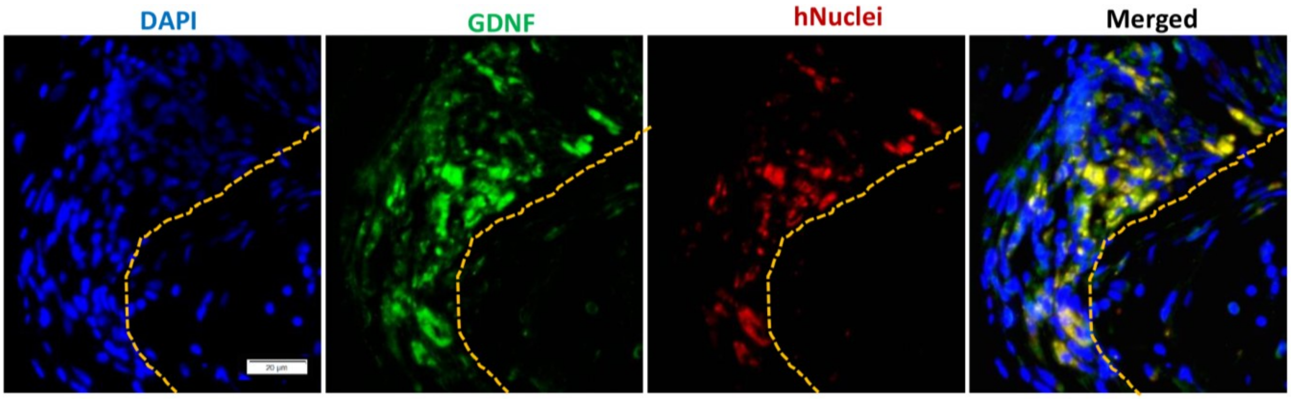

Supplemental Fig. 7. The fate of GiSCs integrated in the nerve conduits following implantation *in vivo*. 14 weeks post-injury and implantation, the newly regenerated facial nerves were harvested and cross-sectional cryosections (10µm in thickness) were cut for immunofluorescence (IF) studies on expression of GDNF (green color) and human nuclei (red color). Nuclei were counterstained with 4', 6-diamidino-2-phenylindole (DAPI; blue). Scale bars=20 µm. Images are representative of three independent experiments. hNuclei, human nuclei.

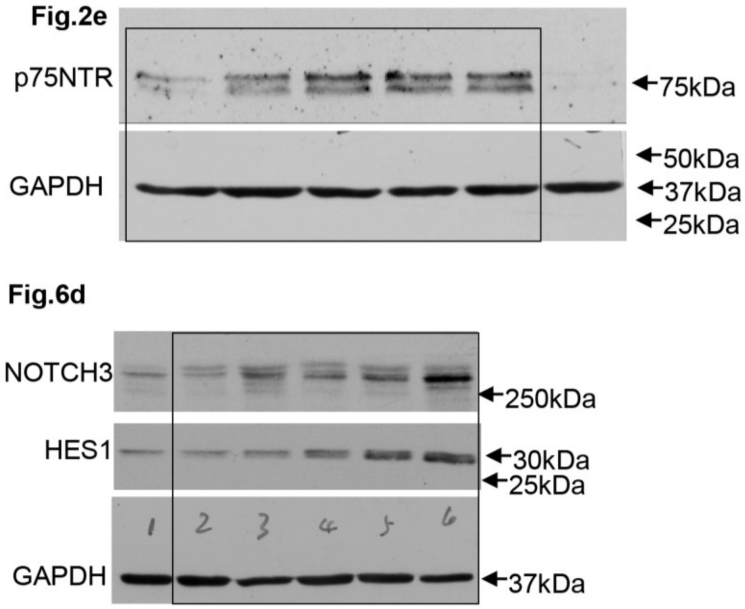

Supplemental Fig. 8. Uncropped Western blotting images for Fig. 2e and Fig. 6d. All blots were derived from the same experiment and processed in parallel, where GAPDH was used as an internal loading control and the size markers were labeled.

Supplemental Table 1. qRT-PCR primer sequences

Gene name		Sequences
CD90	F	5'- AGAGACTTGGATGAGGAG-3'
	R	5'- CTGAGAATGCTGGAGATG-3'
CD73	F	5'- CAGTACCAGGGCACTATCTGG-3'
	R	5'- AGTGGCCCCTTTGCTTTAAT-3'
Col-IA1	F	5'- GTGCTAAAGGTGCCAATGGT-3'
	R	5'- ACCAGGTTCACCGCTGTTAC-3'
VCL	F	5'-TGATGTCATTGCCCTTGC-3'
	R	5'-AGACCTTGAACAACTCCGACTA-3'
β-actin	F	5'-GAGACCTTCAACACCCCAGCC-3'
	R	5'-AATGTCACGCACGATTTCCC-3'
$p75^{NTR}$	F	5'-CCTCATCCCTGTCTATTGCTCC-3'
	R	5'-GTTGGCTCCTTGCTTGTTCTGC-3'
SOX9	F	5'-AGCGAACGCACATCAAGAC-3'
	R	5'-CTGTAGGCGATCTGTTGGGG-3'
ERRFI1	F	5'-ACTCACAGTGAATGGGGTTTG-3'
	R	5'-AGAGAGGGCTTCAGAGATTGG-3'
GDNF	F	5'-AAAGTAGGGCAGGCATGTTG-3'
	R	5'-ATTTCCTGGGAACCTTGGTC-3'
NTF3	F	5'-CCTGCTGGGTAGTGGCTGCG-3'
	R	5'-CATGGCATCCGTGTGGCCGT-3'
TWIST1	F	5'-ACTGGCCTGCAAAACCATAG-3'
	R	5'-GCATTTTACCATGGGTCCTC-3'
NOTCH3	F	5'-ATGCAGGATAGCAAGGAGGA-3'
	R	5'-AAGTGGTCCAACAGCAGCTT-3'
JAG2	F	5'-GTCGTCATCCCCTTCCAGT-3'
	R	5'-CTCCTCATTCGGGGTGGTAT-3'
DLL1	F	5'-CTA CTA CGG AGA GGG CTG CT-3'
	R	5'-CCA GGG TTG CAC ACT TTC C-3'
DLL4	F	5'-AGG CCT GTT TTG TGA CCA AG-3'
	R	5'-GTG CAG GTG TAG CTT CGC T-3'
HES1	F	5'-CGG ACA TTC TGG AAA TGA CA-3'
	R	5'-TAC TTC CCC AGC ACA CTT GG-3'
HEY1	F	5'-TGGATCACCTGAAAATGCTG-3'
	R	5'-CGAAATC CCAAACTCCGATA-3'
GAPDH	F	5'-TCC AAC CTC AGC GTC TTC AC-3'
	R	5'-TGG TGA AGA CGC CAG TGG A-3'
