# Supplementary material for: Harnessing 3D collagen hydrogel-directed conversion of human GMSCs into SCP-like cells to generate functionalized nerve conduits
Source: NPJ Regen Med. 2021 Sep 30;6:59. doi: 10.1038/s41536-021-00170-y (PMC8484485; doi:10.1038/s41536-021-00170-y)
Supplement: Supplementary file 2 — Reporting Summary [file 41536_2021_170_MOESM2_ESM.pdf]

nature research

Corresponding author(s): Anh D. Le

Last updated by author(s): Aug 28, 2021

Reporting Summary

Nature Research wishes to improve the reproducibility of the work that we publish. This form provides structure for consistency and transparency in reporting. For further information on Nature Research policies, see our Editorial Policies and the Editorial Policy Checklist.

Statistics

For all statistical analyses, confirm that the following items are present in the figure legend, table legend, main text, or Methods section.

n/a	Confirmed	
☐	☒	The exact sample size (n) for each experimental group/condition, given as a discrete number and unit of measurement
☐	☒	A statement on whether measurements were taken from distinct samples or whether the same sample was measured repeatedly
☐	☒	The statistical test(s) used AND whether they are one- or two-sided<br>*Only common tests should be described solely by name; describe more complex techniques in the Methods section.*
☒	☐	A description of all covariates tested
☐	☒	A description of any assumptions or corrections, such as tests of normality and adjustment for multiple comparisons
☐	☒	A full description of the statistical parameters including central tendency (e.g. means) or other basic estimates (e.g. regression coefficient) AND variation (e.g. standard deviation) or associated estimates of uncertainty (e.g. confidence intervals)
☐	☒	For null hypothesis testing, the test statistic (e.g. F, t, r) with confidence intervals, effect sizes, degrees of freedom and P value noted<br>*Give P values as exact values whenever suitable.*
☒	☐	For Bayesian analysis, information on the choice of priors and Markov chain Monte Carlo settings
☒	☐	For hierarchical and complex designs, identification of the appropriate level for tests and full reporting of outcomes
☒	☐	Estimates of effect sizes (e.g. Cohen's d, Pearson's r), indicating how they were calculated

Our web collection on statistics for biologists contains articles on many of the points above.

Software and code

Policy information about availability of computer code

Data collection	Excell; cellSens Dimension software;
Data analysis	Excell Data Analysis; SPSS Statistics version 18.0 ; edgeR (R package; StringTie v.1.3.3b software; DAVID Gene Functional Classification Tool

For manuscripts utilizing custom algorithms or software that are central to the research but not yet described in published literature, software must be made available to editors and reviewers. We strongly encourage code deposition in a community repository (e.g. GitHub). See the Nature Research guidelines for submitting code & software for further information.

Data

Policy information about availability of data

All manuscripts must include a data availability statement. This statement should provide the following information, where applicable:

- Accession codes, unique identifiers, or web links for publicly available datasets
- A list of figures that have associated raw data
- A description of any restrictions on data availability

Provide your data availability statement here.

Field-specific reporting

Please select the one below that is the best fit for your research. If you are not sure, read the appropriate sections before making your selection.

☒ Life sciences ☐ Behavioural & social sciences ☐ Ecological, evolutionary & environmental sciences

For a reference copy of the document with all sections, see nature.com/documents/nr-reporting-summary-flat.pdf

Life sciences study design

All studies must disclose on these points even when the disclosure is negative.

Sample size	Two or more biological replicates were carried out for each experiment.
Data exclusions	N/A
Replication	In vitro data were obtained from three independent experiments.
Randomization	Rats were randomly allocated into different treatment groups and the control group.
Blinding	Blinded assessments were performed for all in vivo studies.

Reporting for specific materials, systems and methods

We require information from authors about some types of materials, experimental systems and methods used in many studies. Here, indicate whether each material, system or method listed is relevant to your study. If you are not sure if a list item applies to your research, read the appropriate section before selecting a response.

Materials & experimental systems

n/a	Involved in the study	
☐	☒	Antibodies
☐	☒	Eukaryotic cell lines
☒	☐	Palaeontology and archaeology
☐	☒	Animals and other organisms
☒	☐	Human research participants
☒	☐	Clinical data
☒	☐	Dual use research of concern

Methods

n/a	Involved in the study	
☒	☐	ChIP-seq
☐	☒	Flow cytometry
☒	☐	MRI-based neuroimaging

Antibodies

Antibodies used	For IF: mouse anti-p75 (5 µg/mL, Sigma, St. Louis, Missouri, USA), rabbit anti-SOX-9 (5µg/mL, Cell Signaling Tech, Danvers, Massachusetts, USA), rabbit anti-HES1 (5µg/mL, Cell Signaling Tech, Danvers, Massachusetts, USA), mouse anti-SOX10 (5µg/mL, R & D System, Minneapolis, Minnesota, USA), rabbit anti-S-100beta (5µg/mL, Boster Biological Tech, Pleasanton, California), rabbit anti-Notch3 (2.5µg/mL, Abcam, Cambridge, Massachusetts, USA), rabbit anti-BDNF (2.5µg/mL, Abcam, Cambridge, Massachusetts, USA), rabbit anti-GDNF (2.5µg/mL, Abcam, Cambridge, Massachusetts, USA), rabbit anti-NGF (2.5µg/mL, Abcam, Cambridge, Massachusetts, USA), mouse anti-vinculin (5 µg/mL, Sigma, St. Louis, Missouri, USA), TRITC-phalloidin (0.5 µg/mL, Sigma, St. Louis, Missouri, USA), mouse anti-neurofilament (5µg/mL, BioLegend, San Diego, California, USA), goat anti-rabbit IgG, Alexa Fluor 488, goat anti-rabbit IgG, Alexa Fluor 594, goat anti-mouse IgG, Alexa Fluor 488, goat anti-mouse IgG, Alexa Fluor 594, or normal anti-rabbit, normal anti-mouse IgG, Alexa Fluor 488 , Alexa Fluor 594 (2.5 µg/mL, BioLegend, San Diego, California, USA). For Western blot: rabbit anti-p75 (1µg/mL, Cell Signaling Tech, Danvers, Massachusetts, USA), rabbit anti-HES1 (1µg/mL, Cell Signaling Tech, Danvers, Massachusetts, USA), rabbit anti-GAPDH (1 µg/mL, Cell Signaling Tech, Danvers, Massachusetts, USA), rabbit anti-Notch3 (1µg/mL, Abcam, Cambridge, Massachusetts, USA).
Validation	Each antibody was validated according to manufacturers' protocols.

Eukaryotic cell lines

Policy information about cell lines

Cell line source(s)	Human gingiva derived mesenchymal stem cells (GMSCs) were isolated from healthy subjects who underwent oral surgery procedures and approved by IRB of Upenn.

Authentication	Human gingiva derived mesenchymal stem cells (GMSCs) were isolated from healthy subjects who underwent oral surgery procedures and approved by Upenn IACUC.
Mycoplasma contamination	All cell strains were tested negative for mycoplasma contamination by Upenn Cellcenter Services.
Commonly misidentified lines (See ICLAC register)	N/A

Animals and other organisms

Policy information about studies involving animals; ARRIVE guidelines recommended for reporting animal research

Laboratory animals	Sprague-Dawley rats, female, aged 6-8 weeks old
Wild animals	N/A
Field-collected samples	N/A
Ethics oversight	All animal procedures were approved by the Institutional Animal Care and Use Committee (IACUC) of University of Pennsylvania (Protocol No. 805451).

Note that full information on the approval of the study protocol must also be provided in the manuscript.

Flow Cytometry

Plots

Confirm that:

☒ The axis labels state the marker and fluorochrome used (e.g. CD4-FITC).

☒ The axis scales are clearly visible. Include numbers along axes only for bottom left plot of group (a 'group' is an analysis of identical markers).

☒ All plots are contour plots with outliers or pseudocolor plots.

☒ A numerical value for number of cells or percentage (with statistics) is provided.

Methodology

Sample preparation	2D-cultured GMSCs or GMSCs recovered from 3D-collagen gels via digestion with collagenase I were immunostained with specific antibodies for human CD90 (THY1) (1:200, BioLegend) or p75(1:200, Sigma) or an isotype control, followed by incubation with Alexa Fluor 488-conjugated secondary antibodies.
Instrument	BD FACSCalibur Flow Cytometer
Software	FlowJo software
Cell population abundance	*Describe the abundance of the relevant cell populations within post-sort fractions, providing details on the purity of the samples and how it was determined.*
Gating strategy	*Describe the gating strategy used for all relevant experiments, specifying the preliminary FSC/SSC gates of the starting cell population, indicating where boundaries between "positive" and "negative" staining cell populations are defined.*

☒ Tick this box to confirm that a figure exemplifying the gating strategy is provided in the Supplementary Information.
